# Quality of life in long-term breast cancer survivors in Sub-Saharan Africa: the African Breast Cancer–Disparities in Outcomes study

**DOI:** 10.1007/s11764-024-01693-1

**Published:** 2024-12-10

**Authors:** Pauline Boucheron, Valerie McCormack, Allen Naamala, Chris Sule Oyamienlen, Johanna Pontac, Agnes Kaggwa, Teopista Nakazibwe, Ann Nteziryayo, Esther Ezeigbo, Kingsley Iwuoha, Selma Elishi, Moses Galukande, Angelica Anele, Annelle Zietsman, Milena Foerster, Joachim Schüz, Isabel dos-Santos-Silva

**Affiliations:** 1 International Agency for Research On Cancer (IARC/WHO), Environment and Lifestyle Epidemiology Branch, Lyon, France; 2 College of Health Sciences, Makerere University, Kampala, Uganda; 3 Federal Medical Centre, Owerri, Nigeria; 4 AB May Cancer Centre, Windhoek Central Hospital, Windhoek, Namibia; 5 Department of Non-Communicable Disease Epidemiology, London School of Hygiene and Tropical Medicine (LSHTM), London, UK

**Keywords:** Quality of life, Breast cancer survivors, Survivorship, General population, Sub-Saharan Africa, WHOQOL-BREF

## Abstract

**Purpose:**

In Sub-Saharan Africa (SSA), breast cancer survivors’ (BCS) quality of life (QoL) remains understudied. We compared QoL in BCS to cancer-free (CF) women across SSA settings with different levels of development, healthcare systems, ethnic compositions, and HIV prevalence.

**Methods:**

In 2022–2023, all 5 + year BCS from the African Breast Cancer–Disparities in Outcomes study and age-matched CF women from the community setting answered the WHOQOL-BREF questionnaire in Namibia, Nigeria, and Uganda. For each WHOQOL-BREF domain and general item, we estimated BCS-CF adjusted mean differences (AMD) in scores in absolute percentage points (p.p.).

**Results:**

Overall, 862 women (BCS 357 vs. CF 505) (mean age ± SD, 57.5 ± 12.5 vs. 55.1 ± 11.2; tertiary education, 30% vs. 20%) were included. BCS reported higher/better overall QoL (AMD (95% CI) 10.65 p.p. (7.56, 13.74), i.e., ~ 40% of a change in QoL category), general health (5.84 (2.71, 8.98)), psychological (3.46 (1.43, 5.49)), social relationships (3.94 (1.25, 6.63)), and environment scores (4.27 (1.88, 6.67)) than CF women. There was no BCS-CF difference for physical health in all settings (1.52 (− 0.82, 3.87)), psychological health in Namibian Black women (0.58 (− 2.90, 4.06)), and social relationships in Nigeria (− 0.33 (− 5.81, 5.14)). BCS reported both lower energy and sexual life satisfaction than CF women.

**Conclusions:**

In SSA, long-term BCS have slightly better QoL than CF women except for physical health. Areas for which BCS scored lower or similar than CF women may indicate BCS’ QoL issues.

**Implications for Cancer Survivors:**

BCS-only studies using cancer-specific QoL questionnaires are needed to better characterize BCS’ QoL in SSA.

**Supplementary Information:**

The online version contains supplementary material available at 10.1007/s11764-024-01693-1.

## Introduction

Breast cancer (BC) is the most common cancer in women worldwide, with over 2.3 million new cases in 2020 [1]. Its incidence (in absolute numbers) is rising in low- and middle-income countries (LMIC). Globally, ~ 7.8 million women alive in 2020 were diagnosed with BC in the previous 5 years, of which ~ 430,000 (5.5%) were in Africa [2]. Despite only half of BC patients surviving to 5 years in Sub-Saharan Africa (SSA), this population of BC survivors (BCS) is expected to grow with improvements in early detection and management of BC fostered by the World Health Organization (WHO) Global Breast Cancer Initiative [3, 4].

Quality of life (QoL), defined by the WHO as “individuals’ perceptions of their position in life in the context of the culture and value systems in which they live and in relation to their goals, expectations, standards and concerns,” is an important aspect of cancer survivorship which encompasses physical, psychological, and social well-being [5–7]. BCS are at an increased risk of experiencing specific chronic treatment- or disease-related side effects, such as lymphedema, the physical and psychological impacts of lumpectomy or mastectomy with or without reconstruction, cognitive issues, osteoporosis, cardiac disease, induced menopause, and altered sexual life [8]. Impaired social relationships or financial hardship have also been documented [6, 9]. Furthermore, uncertainties about the future and concerns related to fear of late recurrences or premature death may add to their burden during the survivorship period.

In SSA, QoL of BCS may also differ from other settings, influenced by differences in socioeconomic, cultural, and healthcare characteristics [10]. The clinical profile at BC diagnosis also differs. Compared with high-income countries (HIC), age at diagnosis is lower due to the younger age structure of the population; hence, in SSA, fertility and sexual life issues may have higher impact on BCS; HIV prevalence is higher, and advanced stage at diagnosis and more invasive or suboptimal treatments are frequent, leading to potentially higher treatment-related toxicity [11, 12]. Notably, mastectomy with axillary lymph node dissection constitutes the most common surgery and is sometimes carried out by non-specialists without breast reconstruction and variable access to prostheses. This may trigger the development of BC-related lymphedema; aesthetic outcomes may also be suboptimal [13]. After initial treatment, rehabilitation care is limited, and BC social and financial consequences can be challenging for families as diagnosis and treatment costs are often paid out-of-pocket [14].

Most studies evaluating BCS’ QoL in SSA included BCS still receiving initial treatment or used cancer-specific questionnaires to identify potential QoL issues [15–20]. While these questionnaires enable in-depth QOL assessment in BCS, to disentangle setting-specific from disease-specific influences on long-term QoL and to assess the extent to which BCS regain the QoL they had prior to diagnosis, another approach is to administer a generic (i.e., not cancer-specific) QoL questionnaire to both BCS and a comparison group of cancer-free (CF) women from the same community settings.

Using data from a QoL survey performed in three countries participating in the African Breast Cancer–Disparities in Outcomes (ABC-DO) study, we aimed to (i) evaluate 5 + year BCS’ general QoL in SSA, (ii) compare their QoL to that of an age-matched sample of CF women, and (iii) investigate whether BCS-CF differences in QoL were due to characteristics of these two groups.

## Methods

### Study design and participants

We performed a QoL survey in 5 to 9-year post-diagnosis BCS participating in the ABC-DO study—a hospital-based prospective cohort of BC patients whose protocol is published—and in CF women from the general community setting, in Namibia (Southern Africa), Nigeria (West Africa), and Uganda (East Africa), three SSA countries of different contexts, cultures, and human development index (HDI) [21]. BCS were women aged 18 years or above diagnosed with incident BC between December 13, 2013, and March 31, 2017, who were recruited when they presented at one of ABC-DO study hospitals. All ABC-DO cohort members were actively followed up trimonthly by phone which minimized losses to follow-up [22]. For the present study, we performed a one-off QoL assessment in all 357 ABC-DO BCS who were alive and still being followed after the end of the lockdowns associated with the COVID pandemic.

The CF group was frequency-matched to the BCS on age at QoL interview in each setting, with 150 Black women recruited per country, and, in Namibia, another 50 non-Black (i.e., White or Mixed ancestry) women to match the ABC-DO BCS race distribution observed in this multi-racial setting. CF women were recruited from (i) marketplaces and churches in Owerri (Igbo state, Nigeria) where most Nigerian BCS resided, (ii) marketplaces and relatives of outpatients in Kampala and surrounding districts (Wakiso, Jinja, and Mukono) in Uganda, and (iii) shopping malls and marketplaces in Windhoek, Namibia’s capital, where respectively 60% and 98% of the Namibian Black and non-Black BCS resided. In each recruitment site, a study stand was set up; women approached interviewers voluntarily and eligible women who consented to participate were interviewed. Those below 18 years with a history of cancer or who were currently pregnant were excluded. CF participants were compensated for their time (i.e., the equivalent in local currency of ~ US $8 to $10). All interviews were completed between 5th February 2022 and 17th March 2023 (CF, < 1 month in all settings; BCS, 2 months (Nigeria) to 10 months (Namibia)).

### Data collection

QoL information was collected during a one-off interview (i) at BCS’ last ABC-DO follow-up phone call and (ii) face-to-face at recruitment for CF women. To allow for BCS-CF comparisons, we used the WHOQOL-BREF questionnaire (English version)—the short version of the WHOQOL-100, a validated cross-cultural questionnaire developed by the WHO to measure general QoL (i.e., not cancer-specific) [23, 24]. ABC-DO interviewers were trained in WHOQOL-BREF, which has shown excellent psychometric properties to evaluate QoL and comprises 26 questions, one for each of the 24 facets of the WHOQOL-100 covering 4 domains (i.e., physical health, psychological, social relationships, and environment), plus two questions measuring overall appreciation of general health (QOL_gh_) and overall QoL (QOL_ov_) [25, 26]. Each of the 26 questions is answered using a 5-point Likert scale (e.g., very dissatisfied to very satisfied, not at all to completely, or never to always), respectively, coded from 1 (lowest QoL) to 5 (highest QoL) (notably reversing Qs 3, 4, and 26), which takes ~ 20 min to complete [27]. Participants could opt not to answer a question. Information on potential correlates of QoL (i.e., sociodemographic characteristics and comorbidities) was collected once (i) at recruitment into ABC-DO for BCS and (ii) concomitantly with the QoL interview for CF participants.

### Statistical analysis

We followed the recommendations from the user’s manual to calculate, for each woman’s outcome (i.e., QOL_ov_, QOL_gh_, and each of the four QoL domains) and among the non-missing scores, the arithmetic mean of the constituent question scores, and scaled them to 0–100 so that these are expressed as a percentage [28]. For each question, answers were non-missing for over 99% of women, except for sexual life satisfaction (i.e., Q21), which was missing for 11% of women overall, with the highest percentages missing in BCS and in Nigeria (% missing: BCS, 20%; CF, 12%) and Uganda (39%:18%, respectively). Thus, scores for the social relationships domain were calculated without this question and, where non-missing, this question was reported separately.

For aim (i), we examined the score distribution of domain-specific items and outcomes by setting and group (i.e., BCS and CF). We estimated Cronbach’s alpha to evaluate WHOQOL-BREF’s internal consistency and described between-domain correlations using Pearson’s correlation coefficient. For aim (ii), we fitted, for each domain-specific item and each outcome, linear regression models adjusted for design variables (i.e., age at QoL interview in 5-year bands, country/race, and interviewer) to investigate BCS-CF adjusted mean differences (AMD) in scores in absolute percentage points (p.p.), overall and stratified by study setting. As sensitivity analysis, we restricted the analysis to HIV-negative women. For aim (iii), we identified correlates (i.e., sociodemographic characteristics and comorbidities) of each outcome, added these one by one to the adjusted models, and described their respective effect on BCS-CF AMD. We checked assumptions of linear regression models graphically (i.e., distribution of residuals by group). All analyses were performed using STATA v17.

## Results

### Characteristics of the study population

Overall, 862 women (BCS, 357 (41%); CF, 505 (59%)) participated in this QoL survey, including 283 (33%) Namibian Black (BCS, 136; CF, 147) and 107 (12%) non-Black (58:49, respectively) women, 227 (26%) Nigerian (75:152), and 246 (29%) Ugandan (88:157) women (Table 1). In BCS, mean time since diagnosis was 6.7 years (± 0.7, min–max = 5.0–8.7). BCS had slightly higher prevalence of early-stage diagnosis but similar SEP indicators than ABC-DO women who were potential BCS but lost to follow-up (*n* = 110, % of full ABC-DO cohort: 6% of Black and 13% of non-Black Namibians, 6% of Nigerians, and 11% of Ugandans) (Additional Table 1). BCS were on average ~ 2 years older than CF women (mean age, 57.5 vs. 55.1 years, driven by older average age in the 65 + age group), were more educated (higher education, 30% vs. 20%), and more likely to have ever drunk alcohol (55% vs. 45%) or to be married (52% vs. 46%). While the prevalence of HIV positivity among BCS was similar to that of CF women (13% vs. 16% in Namibian Black and 13% vs. 12% in Uganda, respectively; ≤ 3% for Namibian non-Black and Nigerian BCS and CF women), the prevalence of obesity and of other comorbidities were lower in BCS than in CF women (28% vs. 36% and 42% vs. 50%, respectively), the latter being driven by lower prevalence of hypertension (35% vs. 42%) and diabetes (5% vs. 13%) in BCS.

**Table 1:** Characteristics of the ABC-DO breast cancer survivor subset and the group of cancer-free women who completed the QoL survey

		Namibia non-Black		Namibia Black		Nigeria		Uganda		Overall		
		Breast cancer survivors (*n* = 58)	Cancer-free women (*n* = 49)	Breast cancer survivors (*n* = 136)	Cancer-free women (*n* = 147)	Breast cancer survivors (*n* = 75)	Cancer-free women (*n* = 152)	Breast cancer survivors (*n* = 88)	Cancer-free women (*n* = 158)	Breast cancer survivors (*n* = 357)	Cancer-free women (*n* = 505)	*p* value
		*N* (col %)	*N* (col %)	*N* (col %)	*N* (col %)	*N* (col %)	*N* (col %)	*N* (col %)	*N* (col %)	*N* (col %)	*N* (col %)	
Demographics												
Time since diagnosis	Mean time, in years (SD)	6.9 (0.6)	–	6.9 (0.6)	–	6.2 (0.8)	–	6.6 (0.7)	–	6.7 (0.7)	–	
Age at QoL interview	Mean age (SD)	62.5 (12.2)	61.5 (11.1)	58.0 (13.0)	57.5 (11.6)	54.8 (10.9)	53.2 (10.3)	55.9 (12.2)	52.7 (10.6)	57.5 (12.5)	55.1 (11.2)	0.003
Education	None	0 (0.0)	1 (2.0)	13 (9.6)	12 (8.2)	3 (4.0)	3 (2.0)	8 (9.1)	11 (7.0)	24 (6.7)	27 (5.3)	0.01
	Primary school	2 (3.4)	8 (16.3)	45 (33.1)	45 (30.6)	11 (14.7)	32 (21.1)	38 (43.2)	78 (49.7)	96 (26.9)	163 (32.3)	
	Secondary/high school	28 (48.3)	26 (53.1)	52 (38.2)	66 (44.9)	27 (36.0)	63 (41.4)	22 (25.0)	58 (36.9)	129 (36.1)	213 (42.2)	
	Technical	15 (25.9)	5 (10.2)	9 (6.6)	14 (9.5)	13 (17.3)	11 (7.2)	13 (14.8)	10 (6.4)	50 (14.0)	40 (7.9)	
	University	13 (22.4)	9 (18.4)	17 (12.5)	10 (6.8)	21 (28.0)	43 (28.3)	7 (8.0)	0 (0.0)	58 (16.2)	62 (12.3)	
Residential area*	Rural	1 (1.7)	0 (0.0)	54 (39.7)	2 (1.4)	27 (36.0)	43 (28.3)	60 (68.2)	108 (68.8)	142 (39.8)	153 (30.3)	0.35
Marital status	Married	34 (58.6)	21 (42.9)	53 (39.0)	31 (21.1)	57 (76.0)	126 (82.9)	43 (48.9)	56 (35.7)	187 (52.4)	234 (46.3)	0.08
No. children at home	Median no. (IQR)	0 (0, 2)	1 (0, 2)	2 (1, 3)	2 (1, 3)	3 (1, 4)	4 (2, 5)	2 (1, 4)	2 (0, 4)	2 (0, 4)	2 (0, 4)	0.26
Comorbidities												
HIV status*	Positive	2 (3.4)	1 (2.0)	18 (13.2)	24 (16.3)	2 (2.7)	0 (0.0)	11 (12.5)	19 (12.1)	33 (9.2)	44 (8.7)	0.80
BMI (kg/m^2^)	BMI ≥ 30 +	27 (46.6)	20 (40.8)	36 (26.5)	42 (28.6)	20 (26.7)	69 (45.4)	17 (19.3)	53 (33.8)	100 (28.0)	183 (36.2)	0.01
	Mean BMI (SD)	29.4 (6.8)	29.0 (6.9)	27.3 (6.4)	27.5 (5.8)	27.4 (4.7)	30.3 (6.3)	26.6 (4.7)	28.0 (5.5)	27.5 (5.8)	28.6 (6.1)	0.01
Other comorbidities	Hypertension	27 (46.6)	26 (53.1)	55 (40.4)	80 (54.4)	26 (34.7)	58 (38.2)	16 (18.2)	47 (29.9)	124 (34.7)	211 (41.8)	0.04
	Other CVD	6 (10.3)	11 (22.4)	4 (2.9)	6 (4.1)	4 (5.3)	1 (0.7)	1 (1.1)	4 (2.5)	15 (4.2)	22 (4.4)	0.91
	Diabetes	3 (5.2)	5 (10.2)	6 (4.4)	26 (17.7)	5 (6.7)	15 (9.9)	5 (5.7)	18 (11.5)	19 (5.3)	64 (12.7)	0.0004
	Asthma/COPD	6 (10.3)	3 (6.1)	9 (6.6)	18 (12.2)	2 (2.7)	5 (3.3)	2 (2.3)	3 (1.9)	19 (5.3)	29 (5.7)	0.89
	Tuberculosis	2 (3.4)	0 (0.0)	10 (7.4)	10 (6.8)	0 (0.0)	0 (0.0)	1 (1.1)	2 (1.3)	13 (3.6)	12 (2.4)	0.20
	Any of the above	34 (58.6)	29 (59.2)	66 (48.5)	93 (63.3)	67 (44.1)	20 (22.7)	20 (22.7)	63 (40.1)	150 (42.0)	252 (49.9)	0.02
Tobacco (smoking/smokeless)*	Ever user	20 (34.5)	13 (26.5)	25 (18.4)	24 (16.3)	0 (0.0)	3 (3.4)	3 (3.4)	2 (1.3)	48 (13.4)	39 (7.7)	0.30
Alcohol	Ever drunk	35 (60.3)	28 (57.1)	71 (52.2)	77 (52.4)	89 (58.6)	44 (50.0)	44 (50.0)	35 (22.3)	196 (54.9)	229 (45.3)	0.01

*ABC-DO* African Breast Cancer–Disparities in Outcomes, *BMI* body mass index, *COPD* chronic obstructive pulmonary disease, *CVD* cardiovascular disease, *IQR* interquartile range, *QoL* quality of life, *SD* standard deviation

^*^Chi-square tests restricted to Nigeria and Uganda (residential area), Namibia women and Uganda (HIV status), and Namibia (tobacco), due to the very low prevalence of these factors in the other settings

### Performance of the WHOQOL-BREF questionnaire in the study population

Internal consistency was acceptable for all QoL domains, with Cronbach’s alpha coefficients ranging from 0.62 for the social relationships domain to 0.86 for the physical health domain (Additional Table 2). All domain scores were positively correlated with both the QOL_gh_ and QOL_ov_ scores and with each other (Additional Table 2). QOL_gh_ and QOL_ov_ were most strongly correlated with both the psychological (Pearson correlation coefficient (*r*) = 0.68 and 0.67, respectively) and physical health domains (*r* = 0.66 and *r* = 0.59, respectively) and the least with the social relationships domain (*r* = 0.42 and *r* = 0.43, respectively). The psychological domain correlated most strongly with all others (physical health, 0.77; environment, 0.67; social relationships, 0.62).

### WHOQOL-BREF QoL scores in BCS

**Fig. 1:**
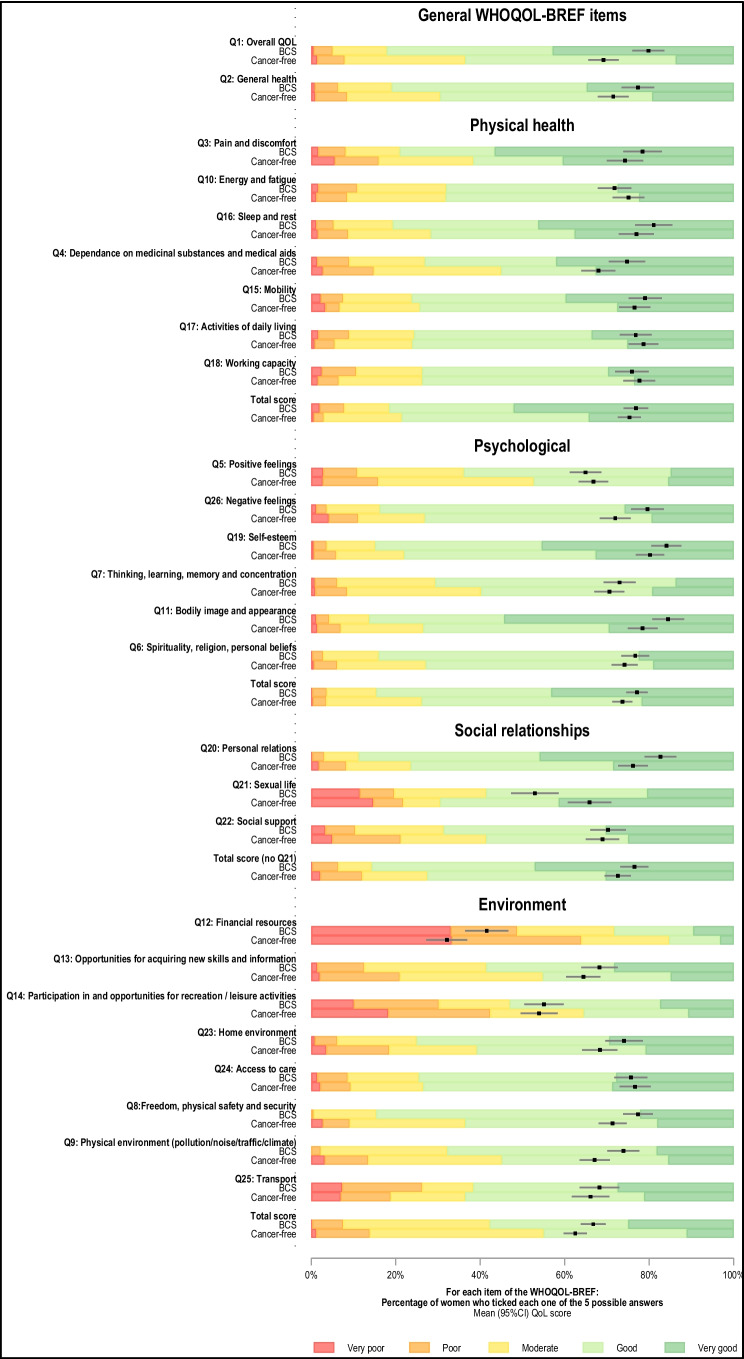
Distribution of WHOQOL-BREF item scores by QoL domain, in ABC-DO breast cancer survivors and cancer-free women. BCS, ABC-DO breast cancer survivors; QoL, quality of life. NB: Adjusted mean scores (95% CI) obtained from linear regression models adjusted for interviewer (categorical), country (Namibia, Nigeria, Uganda), race (Black, non-Black), and age (< 45, 45–49, 50–54, 55–59, 60–64, and 65 + years) and predicted for a woman in the age category 55–59 years, except for Namibian non-Black women (the age category 60–64 years was used for these women because they were, on average, older than those in other settings), interviewed by the interviewer whose adjusted mean QoL score was closest to the overall adjusted mean QoL score

Overall, BCS had good QOL_ov_ and QOL_gh_ scores (mean score (95% CI) 80% (77, 82) and 77% (75, 79), respectively) (Fig. 1, Additional Table 3). BCS had high QoL scores in the physical health (76 (74, 78)), psychological (76 (74, 77)), and social relationships (i.e., calculated without considering sexual life satisfaction) (77 (75, 79)) domains, while that of the environment domain was 12 p.p. lower (65 (64, 67)). In each domain, less than 10% of BCS reported poor or very poor QoL. However, there were between-settings disparities in QoL, with scores being constantly highest in Namibia (and within Namibia, in non-Black women) and lowest in Uganda.

### Differences in general WHOQOL-BREF items between BCS and CF groups

**Fig. 2:**
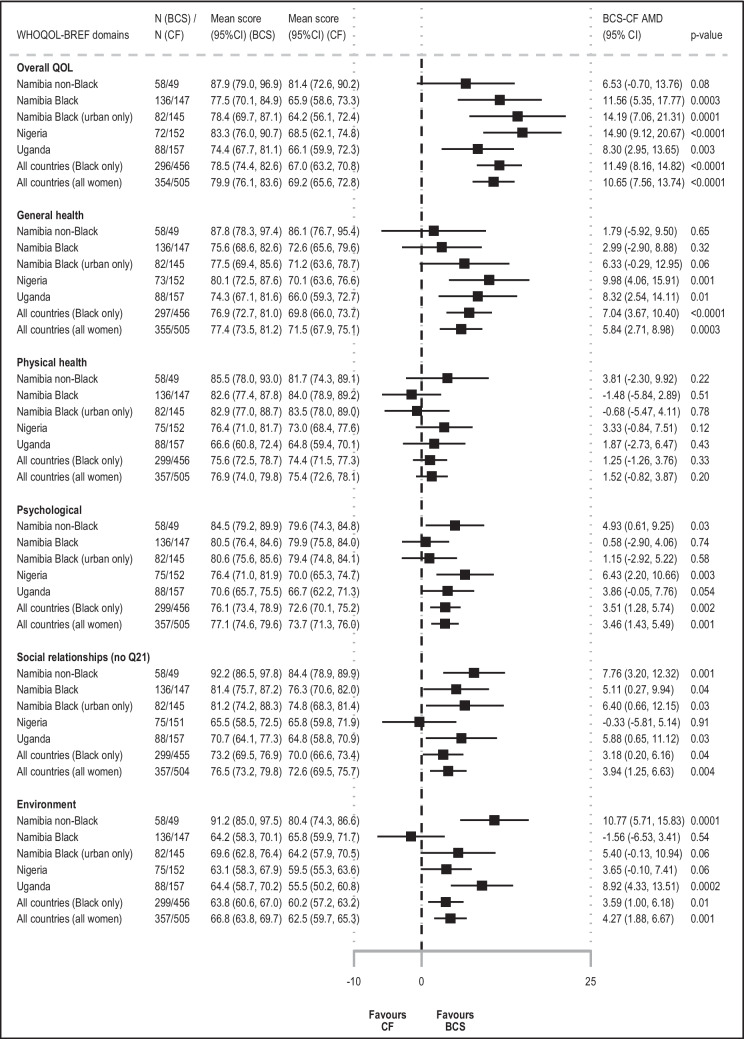
Adjusted mean differences (AMD) in WHOQOL-BREF domain scores between breast cancer survivors (BCS) and cancer-free (CF) women, by country and race. AMD, adjusted mean difference; BCS, breast cancer survivors; CF, cancer-free women; 95% CI, 95% confidence interval; QoL, quality of life. NB1: Absolute BCS-CF adjusted mean difference (AMD) in QoL scores in percentage points (p.p.), with 95% CI, obtained from linear regression models adjusted for interviewer (categorical), country (Namibia, Nigeria, Uganda), race (Black, non-Black), and age (< 45, 45–49, 50–54, 55–59, 60–64, and 65 + years). NB2: Adjusted mean scores (95% CI) predicted for a woman in the age category 55–59 years, except for Namibian non-Black women (the age category 60–64 years was used for these women because they were, on average, older than those in other settings), interviewed by the interviewer whose adjusted mean QoL score was closest to the overall adjusted mean QoL score

Overall, BCS had higher QoL scores than their CF counterparts across all general items/domains of the questionnaire (mean BCS-CF absolute difference in scores: QOL_ov_, + 13 p.p.; QOL_gh_, + 7 p.p.; from + 4 p.p. (physical health) to + 8 p.p. (social relationships) for WHOQOL-BREF domains). Upon adjustment for design variables, BCS-CF differences in QOL_ov_ and QOL_gh_ were slightly attenuated (AMD (95% CI) 10.65 p.p. (7.56, 13.74) and 5.84 p.p. (2.71, 8.98), respectively) (Fig. 2). While BCS’ QOL_ov_ scores were higher in every setting (min, + 7% in Namibian non-Black women; max, + 15% in Nigerians), there was no evidence of a BCS-CF difference in QOL_gh_ in Namibian non-Black women (range, from + 6% in Namibian urban Black women to + 10% in Nigerians). Results were similar when restricting to HIV-negative women (Additional Fig. 1). Both QOL_ov_ and QOL_gh_ increased with increasing educational level and with being married, and, for QOL_ov_, also with increasing BMI. In contrast, both QOL_ov_ and QOL_gh_ were lower in women who had comorbidities, had higher number of children at home, and, for QOL_gh_, also in those who ever used tobacco or resided in rural areas (Additional Table 4). In non-Black Namibians, where BCS were substantially more educated than CF women (technical/university education, 48% vs. 29%, respectively), further adjustment for educational level reduced the magnitude of the BCS-CF AMD in QOL_ov_ score by − 2.3 p.p. (i.e., ~ 10% of a change in QoL category) (Table 1, Additional Fig. 2), but did not affect that in QOL_gh_ score (Additional Fig. 3). BCS-CF AMD in QOL_ov_ and QOL_gh_ scores were marginally changed when adjusting for the other correlates (Additional Figs. 2 & 3).

### Differences in WHOQOL-BREF QoL domains scores between BCS and CF groups

#### Physical health domain

**Fig. 3:**
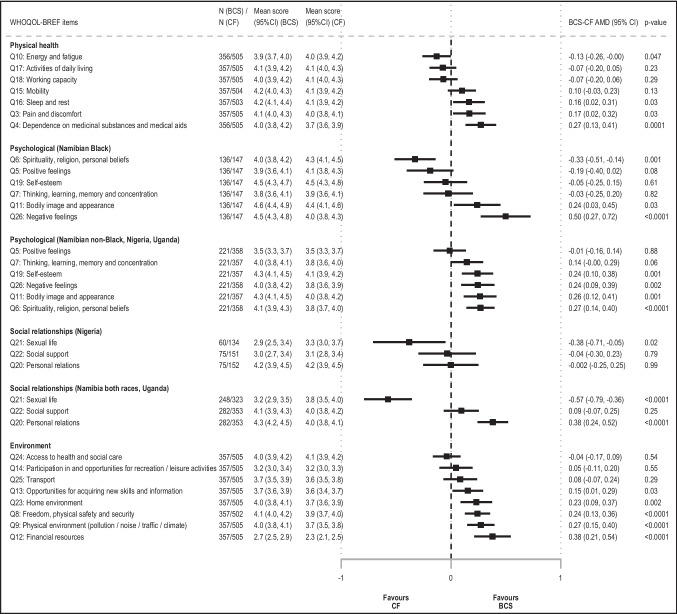
Adjusted mean differences in WHOQOL-BREF domain items between breast cancer survivors (BCS) and cancer-free (CF) women. AMD, adjusted mean difference; BCS, breast cancer survivors; CF, cancer-free women; 95% CI, 95% confidence interval; QoL, quality of life. NB1: Absolute BCS-CF adjusted mean difference (AMD) in item scores, expressed on a scale from 1 (lowest score = worst QoL) to 5 (highest score = best QoL), with 95% CI, obtained from linear regression models adjusted for interviewer (categorical), country (Namibia, Nigeria, Uganda), race (Black, non-Black), and age (< 45, 45–49, 50–54, 55–59, 60–64, and 65 + years). NB2: Adjusted mean scores (95% CI) predicted for a woman in the age category 55–59 years, except for Namibian non-Black women (the age category 60–64 years was used for these women because they were, on average, older than those in other settings), interviewed by the interviewer whose adjusted mean QoL score was closest to the overall adjusted mean QoL score

There was no evidence of BCS-CF differences in physical health scores in any of the settings before or after adjustment for design variables (AMD (95% CI) 1.52 p.p. (− 0.82, 3.87)) (Fig. 2); however, there was large heterogeneity between domain-specific items (Figs. 1 and 3). BCS reported substantially higher levels of fatigue and similar working capacity and ability to perform daily living activities than CF women, but they scored higher in the other items (Fig. 3). Scores were higher in more educated or married women, but were lower in rural women, in those with non-HIV related comorbidities, or in ever tobacco users (Additional Table 4). In each setting, adjusting for any of these correlates changed marginally the magnitude of the BCS-CF AMD in physical health scores (i.e., all < 10% of a change in QoL category) (Additional Fig. 4). Interestingly, in Nigeria and Uganda (i.e., the two countries where this analysis could be undertaken), while urban BCS had higher physical health scores than urban CF women, in rural women, there was no BCS-CF difference (data not shown).

#### Psychological domain

Psychological scores adjusted for design variables remained 4% to 6% higher in BCS than in CF women in Uganda, Nigeria, and Namibia non-Black population groups (AMD (95% CI) 3.86 p.p. (− 0.05, 7.76), 6.43 (2.20, 10.66), and 4.93 (0.61, 9.25), respectively); however, there was no evidence of a BCS-CF difference in Black Namibians (Fig. 2). While in most settings, BCS reported similar levels of positive feelings as CF women and higher scores in the other items, Namibian Black BCS scored lower for both spirituality and positive feelings and did not differ from CF women for cognitive abilities and self-esteem (data not shown). Bodily acceptance was the only item higher in BCS than in CF women in all settings (Fig. 3). Scores increased with both increasing educational level or BMI, and with being married, but were lower in rural women, in those with comorbidities, or in ever tobacco users (Additional Table 4). However, adjusting for each of these correlates changed marginally the magnitude of the BCS-CF difference in psychological domain scores (Additional Fig. 5).

#### Social relationship domain

Social scores adjusted for design variables remained 5% to 8% higher in BCS in most settings (min–max AMD (95% CI) 5.11 p.p. (0.27, 9.94) (Black Namibians); 7.76 (3.20, 12.32) (non-Black Namibians)) driven by higher QoL regarding personal relations (Figs. 2 and 3). In contrast, in Nigeria where social support scores were lower than in the other settings (moderate QoL or lower, ~ 60% in both groups), there was no evidence of a BCS-CF difference in both domain and items scores (Fig. 2, data not shown). Scores increased with both increasing educational level or BMI and with being married (Additional Table 4). However, BCS-CF differences in scores changed marginally when adjusting for any of these correlates (Additional Fig. 6). Among respondents, sexual life satisfaction was substantially lower in BCS than CF women in all settings except Uganda, where scores were low in both groups (poor to very poor QoL: BCS, 32%; CF, 44%) (Figs. 1 and 3, data not shown).

#### Environment domain

Environmental scores adjusted for design variables remained ~ 4% higher in BCS in all settings (min–max AMD (95% CI) 3.65 p.p. (− 0.10, 7.41) (Nigeria); 10.77 (5.71, 15.83) (Namibian non-Black women)), except in Black Namibians, for which the lack of evidence of a BCS-CF difference was driven by rural BCS (urban Black Namibians, 5.40 (− 0.13, 10.94)) (Fig. 2). Both groups scored lowest in financial resources (poor to very poor QoL: BCS 49% vs. CF 64%), opportunities for recreation and leisure activities (30% vs. 42%, respectively), and access to transportation (19% vs. 26%) (Fig. 1). While scores in the latter two items and access to health and social care were similar for BCS and CF women, BCS scored substantially higher in all other domain-specific items (Fig. 3). Environment scores increased with increasing educational level, increasing BMI and being married, but were lower in women who resided in rural areas, in those who were HIV-positive, or in those who ever used tobacco (Additional Table 4). Further adjustment for educational level (i.e., a SEP indicator) reduced the magnitude of the BCS-CF differences in scores among Namibian non-Black women by 2.6 p.p. (~ 10% of a change in QoL category) but only changed marginally the estimates in the other settings (Additional Fig. 7). Adjusting for each one of the other correlates did not affect BCS-CF differences in scores. For all QoL domains, results from adjusted models remained unchanged in sensitivity analysis (Additional Fig. 1).

## Discussion

### Main findings

This survey assessed general QoL of long-term BCS and compared it with that of CF women in three SSA countries. The WHOQOL-BREF questionnaire was reliable and culturally acceptable in this setting, except when inquiring about sexual life in Nigeria and Uganda. Overall, for both general items and most domains, QoL of BCS was good—except for the environment domain for which it was moderate—and tended to be slightly better than that of CF women. However, BCS had similar QoL to that of CF women for the physical health domain in all countries, for psychological health in Namibian black women, and for the social relationship domain in Nigeria, which may point to specific BCS’ QoL issues in these settings.

### Comparison of BCS’ QoL scores with that observed in other studies

Four studies reported long-term BCS’ QoL scores using the WHOQOL-BREF—three were in upper-middle- (Brazil) or high-income settings (Portugal, Israel) and one in SSA (Sudan) [17, 29–31]. In Sudan, at 3 years post-diagnosis, BCS had ~ 5 to 10 p.p. higher QoL scores (i.e., 20% to 50% of a change in QoL category) than ABC-DO BCS in all domains but social relationships, possibly influenced by their younger age (~ 10 years younger than in ABC-DO)—age correlated negatively with QoL scores in all domains except the social domain in our study. However, between-studies interpretation of the social domain was limited as, alike other African settings, inquiring about sexual life was deemed inappropriate, especially in BCS—maybe due to disease representations [16, 32]. Also, in Sudan and aligned with previous work, survivorship duration correlated positively with QoL; however, this was not observed in ABC-DO possibly because BCS were 5 to 9 years post-diagnosis and had all completed initial treatment [17, 33]. In the three other studies, BCS at 5 to 8 years post-diagnosis had lower QoL than ABC-DO BCS in all domains except the environment domain despite residing in higher HDI settings, a known predictor of higher QoL. The higher QoL in ABC-DO BCS is likely partially influenced by a stronger SEP gradient in low survival contexts, expanded upon below [10, 34]. However, differences in HDI may contribute to the between-setting disparities in BCS’ QoL in ABC-DO—QoL scores were substantially lower in both Nigeria and Uganda than in Namibia (HDI_2021_, ~ 0.54 (Nigeria and Uganda) *vs.* 0.62 (Namibia)) [34]. Also, racial disparities in Namibia may result from remaining high inequities in this country [35, 36].

### Comparison of BCS’ QoL domain scores with that of cancer-free women

BC risk and survival both increase with higher SEP [37]. This socioeconomic filtering of survivors is stronger in a low survival context such as SSA where universal health coverage is generally not implemented. In ABC-DO, the survival gap resulted from an accumulation of social disadvantages throughout the BC journey (i.e., from symptom(s) recognition influenced by educational level—a SEP indicator, to better access to diagnosis and treatment with higher SEP), with 3-year mortality rates being 1.5 times higher in women with low SEP than in those with high SEP [3, 11, 14]. In this QoL survey, compared with Namibia, the lower BCS’ sample 5 + years after diagnosis in both Nigeria and Uganda resulted from lower BC survival in these countries, influenced by differences in quality and availability of care—in Namibia, access to diagnosis and treatment is free, while in Nigeria and Uganda, women pay out-of-pocket [3, 11, 14].

Higher SEP is also predictive of higher QoL, in line with our study in which all QoL domains were strongly positively correlated with SEP indicators [10, 38]. Previous work in higher-income settings found similar to lower QoL in long-term BCS (i.e., 5 + years post-diagnosis) than in the general population, including in the environment domain, suggesting a less pronounced SEP gradient in BC survival in these settings [29–31, 33, 38, 39]. In contrast, in Sudan and in our study, BCS had marginally better living conditions than CF women, driven by higher scores in environment domain SEP-related items (i.e., higher financial resources—despite being a matter of concern for all women, better home environment, safer living environment, and better access to information). In our study, SEP marginally explained QoL differences between BCS and CF women. While residual confounding by SEP cannot be ruled out, QoL is a subjective metric (i.e., perception of QoL may differ between BCS and CF women), and in SSA where BC survival is low, BCS may be grateful to be alive, leading them to relativize daily life issues.

Despite BCS reporting higher QoL than CF women, our study identified some areas that require further investigation and for which in-depth interviews with BCS would be warranted. In Namibian Black women and in Nigeria, the psychological and social domains were the only domains for which BCS and CF women had similar scores suggesting that BCS might have faced specific breast cancer-related issues which could not be properly captured by the general QoL questionnaire used in the present study. Although CF women had both higher prevalence of comorbidities and higher levels of pain and dependency on medication than BCS, physical health QoL scores were similar in both groups. This was driven by the substantially higher levels of fatigue reported by BCS, a frequently reported cancer survivorship issue, which may have impacted their ability to perform daily activities or work—two items for which BCS scored slightly lower than CF women [29, 40, 41]. Furthermore, aligned with previous studies, and despite generally better bodily acceptance, BCS reported much lower sexual life satisfaction than CF women, possibly resulting from long-term side-effects of their BC treatment [32, 42–44].

### Strengths and limitations

This study is the first to compare general QoL in both long-term BCS and CF women across settings in SSA. This was rendered possible using the WHOQOL-BREF, a validated cross-cultural questionnaire adapted to perform comparisons with the general population. The risk of misinterpretation of some WHOQOL-BREF questions when orally translated into local languages to non-English speakers was minimized by standardized training of interviewers, which ensured a common understanding of the questions, and on-site supervision by the investigators. Inherent to our study design, group differences in data collection methods may have (i) differentially influenced women responses to WHOQOL-BREF (e.g., trust climate would differ between BCS and CF women), thus possibly contributing to underestimate between-group differences in QoL, and (ii) limited our ability to control for time-dependent correlates of QoL (e.g., marital status, comorbidities, BMI). Few ABC-DO women who were potential BCS but missing may have been alive—BC survival rates are low in SSA—and may have reported a different QoL than BCS; however, their small number would have marginally impacted BCS’ QoL outcomes. Also, recruitment sites (i.e., marketplaces) and the small financial reward may have favored inclusion of CF women with lower SEP indicators, who may not be representative of and may have reported lower QoL outcomes than the general population in each setting. Thus, between-group differences may have been overestimated for QoL outcomes reported as similar or higher in BCS than CF women, but underestimated for those which were lower in BCS.

### Implications

In SSA where inequalities in accessing healthcare remain high, the SEP gradient in the population is likely to influence health and QoL to a much greater extent than in HIC. This study focused on comparing BCS’ QoL to that of women with no history of cancer to assess the extent to which BCS regained the QoL they had prior to their cancer diagnosis. Such comparisons require the use of a generic tool (e.g., WHOQOL-BREF) that can be applied to both cancer and non-cancer individuals as well as consideration of the higher SEP of BCS when choosing the comparison group (e.g., healthy neighbors), particularly so in LMICs. Whenever the focus is on specific cancer-related issues, survivor-only studies are better suited. To assess BCS-specific issues (e.g., lymphedema, sexual health, BC-related financial difficulty, and emotional burden), questionnaires specifically designed for current or former cancer patients (e.g., EORTC QLQ C30 or EORTC Br45)—and not generic ones—should be used [45–47]. Survivorship duration should also be considered when choosing the comparison group (i.e., QoL might change throughout survivorship being lower in survivors still undergoing initial treatment than in long-term survivors).

### Conclusion

In SSA, long-term BCS do not appear to have a worse QoL than CF women from the same communities, and in all domains but physical health, their QoL appears to be marginally better. These findings may be partly explained, although not fully, by SEP disparities in BC risk and BC survival, both of which drive the selection of BCS over time toward a higher SEP. BCS’ QoL also depends on the local context as highlighted by the disparities in QoL scores seen across settings in SSA. The WHOQOL-BREF questionnaire was appropriate and reliable in this context; however, to better understand the QoL of BCS in SSA, BC-specific QoL issues need to be investigated using a cancer-specific questionnaire (e.g., EORTC).

## Supplementary Information

Below is the link to the electronic supplementary material.

Supplementary file1 (DOCX 243 KB)

## Data Availability

The datasets generated during and/or analysed during the current study are available on the study website (https://abc-do.iarc.fr/) or from reasonable request to the study PI (Dr. Valerie McCormack, Prof. Isabel dos Santos Silva).

## Declarations

### Disclaimers

Where authors are identified as personnel of the International Agency for Research on Cancer/World Health Organization, the authors alone are responsible for the views expressed in this article and they do not necessarily represent the decisions, policy, or views of the International Agency for Research on Cancer/World Health Organization. Research reported in this publication was supported by the National Cancer Institute of the National Institutes of Health under Award Number R01CA244559. The content is solely the responsibility of the authors and does not necessarily represent the official views of the National Institutes of Health.
