# Supplementary material for: Quality of life in long-term breast cancer survivors in Sub-Saharan Africa: the African Breast Cancer–Disparities in Outcomes study
Source: J Cancer Surviv. 2024 Dec 10;20(3):870–82. doi: 10.1007/s11764-024-01693-1 (PMC12149336; doi:10.1007/s11764-024-01693-1)
Supplement: Supplementary file 1 — Supplementary file1 (DOCX 243 KB) [file 11764_2024_1693_MOESM1_ESM.docx]

**Table of Contents**

[Additional Table 1. Characteristics of women in the ABC-DO cohort in the settings where the QoL study was conducted (i.e., Namibia, Nigeria, and Uganda), by status at the time of the QoL interview 2](#_Toc161413640)

[Additional Table 2. Correlation* between WHOQOL-BREF General items and QoL domains, and reliability of each QoL domain in the QoL survey 3](#_Toc161413641)

[Additional Table 3. Mean (95% CI) of domain-specific WHOQOL-BREF QoL percentages in breast cancer survivors and cancer-free women, by country and race 4](#_Toc161413642)

[Additional Figure 1. Adjusted mean differences (AMD) in WHOQOL-BREF domain scores between breast cancer survivors (BCS) and cancer-free (CF) women among HIV negative women, by country and race 5](#_Toc161413643)

[Additional Table 4. Correlates of the General QoL items and QoL domains of the WHOQOL-BREF in the QoL survey 6](#_Toc161413644)

[Additional Figure 2. Adjusted mean differences (AMD) in scores between breast cancer survivors (BCS) and cancer-free (CF) women for the overall QoL item of the WHOQOL-BREF, before and after further adjustment for correlates 7](#_Toc161413645)

[Additional Figure 3. Adjusted mean differences (AMD) in scores between breast cancer survivors (BCS) and cancer-free (CF) women for the general health QoL item of the WHOQOL-BREF, before and after further adjustment for correlates 8](#_Toc161413646)

[Additional Figure 4. Adjusted mean differences (AMD) in scores between breast cancer survivors (BCS) and cancer-free (CF) women for the physical health domain of the WHOQOL-BREF, before and after further adjustment for correlates 9](#_Toc161413647)

[Additional Figure 5. Adjusted mean differences (AMD) in scores between breast cancer survivors (BCS) and cancer-free (CF) women for the psychological domain of the WHOQOL-BREF, before and after further adjustment for correlates 10](#_Toc161413648)

[Additional Figure 6. Adjusted mean differences (AMD) in scores between breast cancer survivors (BCS) and cancer-free (CF) women for the social relationships (no Q21)* domain of the WHOQOL-BREF, before and after further adjustment for correlates 11](#_Toc161413649)

[Additional Figure 7. Adjusted mean differences (AMD) in scores between breast cancer survivors (BCS) and cancer-free (CF) women for the environment domain of the WHOQOL-BREF, before and after further adjustment for correlates 12](#_Toc161413650)

# **Additional Table 1. Characteristics of women in the ABC-DO cohort in the settings where the QoL study was conducted (i.e., Namibia, Nigeria, and Uganda), by status at the time of the QoL interview**

|  | | **Full ABC-DO cohort (n=1333)** | **BCS (n=357)** | **Potential BCS but missing (n=110)** | **p-value*** |
| --- | --- | --- | --- | --- | --- |
|  |  | **N (%)** | **N (%)** | **N (%)** |  |
| **Country/ethnicity** | Namibia Black | 388 (29.1) | 136 (85.0) | 24 (15.0) | 0.001 |
|  | Namibia non-Black | 114 (8.6) | 58 (79.5) | 15 (20.5) |  |
|  | Nigeria | 400 (30.0) | 75 (75.8) | 24 (24.2) |  |
|  | Uganda | 431 (32.3) | 88 (65.2) | 47 (34.8) |  |
| **Age at diagnosis** | Mean age, SD | 50.6 (13.6) | 51 (12.4) | 50 (13.4) | 0.34 |
| **Education** | None | 155 (11.6) | 24 (6.7) | 13 (11.8) | 0.33 |
|  | Primary school | 426 (32.0) | 96 (26.9) | 32 (29.1) |  |
|  | Secondary/high school | 466 (35.0) | 129 (36.1) | 39 (35.5) |  |
|  | Technical | 140 (10.5) | 50 (14.0) | 14 (12.7) |  |
|  | University | 146 (11.0) | 58 (16.2) | 12 (10.9) |  |
| **Residential area*** | Urban | 683 (51.2) | 215 (60.2) | 63 (57.3) | 0.58 |
|  | Rural | 650 (48.8) | 142 (39.8) | 47 (42.7) |  |
| **Comorbidities (at recruitment)** | | | | |  |
| **BMI (Kg/m2)** | Mean BMI, SD | 26.4 (5.8) | 28 (5.9) | 26 (5.5) | 0.11 |
| **HIV** | Yes | 116 (8.7) | 33 (9.2) | 8 (7.3) | 0.52 |
|  | No | 1217 (91.3) | 324 (90.8) | 102 (92.7) |  |
| **Other comorbidities** | Yes | 708 (53.1) | 183 (51.3) | 56 (50.9) | 0.95 |
|  | No | 625 (46.9) | 174 (48.7) | 54 (49.1) |  |
| **BC characteristics** | | | | |  |
| **TNM stage at diagnosis** | I | 91 (6.8) | 42 (11.8) | 17 (15.5) | 0.03 |
|  | II | 365 (27.4) | 173 (48.5) | 35 (31.8) |  |
|  | III | 604 (45.3) | 115 (32.2) | 45 (40.9) |  |
|  | IV | 209 (15.7) | 13 (3.6) | 6 (5.5) |  |
|  | Unknown | 64 (4.8) | 14 (3.9) | 7 (6.4) |  |
| **Tumour subtype** | HR+ | 421 (31.6) | 173 (48.5) | 37 (33.6) | 0.79 |
|  | HR- | 158 (11.9) | 47 (13.2) | 9 (8.2) |  |
|  | unknown | 754 (56.6) | 137 (38.4) | 64 (58.2) |  |
| **Any treatment received**** | Yes | 915 (81.4) | 314 (91.3) | 83 (79.8) | 0.07 |
|  | No | 134 (11.9) | 18 (5.2) | 10 (9.6) |  |
|  | Unknown | 75 (6.7) | 12 (3.5) | 11 (10.6) |  |

ABC-DO = African Breast Cancer – Disparities in Outcomes; BC = Breast cancer; BCS = Breast cancer survivors; QoL = Quality of life; SD = Standard deviation.

*p-value obtained from chi-square tests comparing BCS to potential BCS but lost to follow-up. For the BC characteristics, these tests were restricted to non-missing data.

** Among BCS diagnosed with non-metastatic BC who underwent potentially curative treatment: 285 (91%) had surgery (with 81% having mastectomy), 241 (77%) received chemotherapy, 173 (55%) had radiotherapy, and 251 (80%) received endocrine therapy.

# **Additional Table 2. Correlation* between WHOQOL-BREF General items and QoL domains, and reliability of each QoL domain in the QoL survey**

|  | **QOL_gh_^**^** | **QOL_ov_^***^** | **Physical health** | **Psychological** | **Social relationships (no Q21)****** | **Environment** | **Cronbach α coefficient** |
| --- | --- | --- | --- | --- | --- | --- | --- |
| **QOL_gh_^**^** | 1 | - | - | - | - | - | - |
| **QOL_ov_^***^** | 0.72 | 1 | - | - | - | - | - |
| **Physical health** | 0.66 | 0.59 | 1 | - | - | - | 0.86 |
| **Psychological** | 0.68 | 0.67 | 0.77 | 1 | - | - | 0.80 |
| **Social relationships (no Q21)** | 0.42 | 0.43 | 0.51 | 0.62 | 1 | - | 0.62 |
| **Environment** | 0.50 | 0.54 | 0.55 | 0.67 | 0.59 | 1 | 0.85 |

QoL = Quality of life; QOL_gh_ = General health; QOL_ov_ = Overall QoL.

*Pearson’s correlation coefficient.

**two Nigerian BCS had missing QOL_gh_ score.

***Three Nigerian BCS had missing QOL_ov_ score, of which two also had missing QOL_gh_ score.

****Results presented do not take sexual life satisfaction (i.e., Q21) into account, due to the high non-response rate to this question.

# **Additional Table 3. Mean (95% CI) of domain-specific WHOQOL-BREF QoL percentages in breast cancer survivors and cancer-free women, by country and race**

|  | **Namibia non-Black** | | **Namibia Black** | | **Nigeria** | | **Uganda** | | **Overall** | |
| --- | --- | --- | --- | --- | --- | --- | --- | --- | --- | --- |
|  | **Breast Cancer Survivors**  **(n = 58)** | **Cancer-free women**  **(n = 49)** | **Breast Cancer Survivors**  **(n = 136)** | **Cancer-free women**  **(n = 147)** | **Breast Cancer Survivors**  **(n = 75)** | **Cancer-free women**  **(n = 152)** | **Breast Cancer Survivors**  **(n = 88)** | **Cancer-free women**  **(n = 158)** | **Breast Cancer Survivors**  **(n = 357)** | **Cancer-free women**  **(n = 505)** |
| QoL Domain | Mean (95%CI) | Mean (95%CI) | Mean (95%CI) | Mean (95%CI) | Mean (95%CI) | Mean (95%CI) | Mean (95%CI) | Mean (95%CI) | Mean (95%CI) | Mean (95%CI) |
| QOL_gh_ score* | 83.6  (78.5, 88.8) | 83.2  (77.6, 88.8) | 80.0  (76.5, 83.4) | 73.5  (70.2, 76.8) | 76.3  (71.4, 81.3) | 66.8  (63.3, 70.2) | 68.5  (63.8, 73.2) | 65.3  (61.8, 68.8) | 77.0  (74.7, 79.2) | 69.9  (67.9, 71.8) |
| QOL_ov_ score** | 84.9  (80.1, 89.8) | 79.1  (73.8, 84.4) | 82.4  (78.7, 86.0) | 64.6  (61.1, 68.1) | 80.4  (75.5, 85.3) | 65.6  (62.2, 69.1) | 71.0  (66.8, 75.3) | 66.9  (63.7, 70.1) | 79.6  (77.4, 81.8) | 67.0  (65.2, 68.9) |
| Physical health | 82.9  (78.8, 87.1) | 79.9  (75.4, 84.4) | 82.6  (80.0, 85.3) | 79.7  (77.2, 82.3) | 73.2  (69.4, 77.1) | 70.7  (68.0, 73.4) | 63.6  (60.0, 67.2) | 62.4  (59.7, 65.1) | 76.0  (74.1, 77.9) | 71.7  (70.1, 73.2) |
| Psychological | 84.0  (81.1, 86.9) | 79.5  (76.4, 82.7) | 80.9  (78.8, 82.9) | 76.3  (74.3, 78.3) | 71.8  (68.1, 75.5) | 66.1  (63.5, 68.7) | 64.8  (61.7, 67.9) | 64.2  (61.9, 66.5) | 75.5  (73.9, 77.1) | 69.8  (68.4, 71.1) |
| Social relationships (no Q21) | 91.8  (88.7, 95.0) | 84.4  (81.0, 87.9) | 84.7  (82.0, 87.5) | 76.7  (74.0, 79.4) | 65.0  (59.7, 70.3) | 65.7  (62.0, 69.5) | 65.8  (61.7, 69.8) | 60.0  (57.0, 63.1) | 77.1  (74.9, 79.3) | 69.0  (67.1, 70.8) |
| Environment | 87.6  (84.2, 91.0) | 77.0  (73.3, 80.7) | 63.3  (60.4, 66.2) | 60.2  (57.4, 63.0) | 60.6  (57.4, 63.9) | 57.6  (55.3, 59.8) | 58.3  (54.5, 62.1) | 52.4  (49.6, 55.3) | 65.4  (63.5, 67.3) | 58.6  (57.0, 60.2) |

95% CI = 95% confidence interval; QoL = Quality of life; QOL_gh_ = General health; QOL_ov_ = Overall QoL.

*QOL_gh_ score was missing in two Nigerian BCS;

**QOL_ov_ scores were missing in three Nigerian BCS.

# **Additional Figure 1. Adjusted mean differences (AMD) in WHOQOL-BREF domain scores between breast cancer survivors (BCS) and cancer-free (CF) women among HIV negative women, by country and race**

AMD: Adjusted mean difference; BCS = Breast cancer survivors; CF = Cancer-free women; 95% CI = 95% confidence interval; QoL = Quality of life.

NB1: Absolute BCS-CF adjusted mean difference (AMD) in QoL scores in percentage points (p.p.), with 95% CI, obtained from linear models adjusted for interviewer (categorical), country (Namibia, Nigeria, Uganda), race (Black, non-Black) and age (<45, 45-49, 50-54, 55-59, 60-64, and 65+ years).

NB2: Adjusted mean scores (95% CI) predicted for a woman in the age category 55-59 years, except for Namibian non-Black women (the age category 60-64 years was used for these women because they were, on average, older than those in other settings), interviewed by the interviewer whose adjusted mean QoL score was closest to the overall adjusted mean QoL score.

# **Additional Table 4. Correlates of the General QoL items and QoL domains of the WHOQOL-BREF in the QoL survey**

|  | | **QOL_gh_** | | **QOL_ov_** | | **Physical health** | | **Psychological** | | **Social relationships (no Q21)*** | | **Environment** | |
| --- | --- | --- | --- | --- | --- | --- | --- | --- | --- | --- | --- | --- | --- |
|  |  | Absolute mean difference in scores in % (95% CI) | p-value | Absolute mean difference in scores in % (95% CI) | p-value | Absolute mean difference in scores in % (95% CI) | p-value | Absolute mean difference in scores in % (95% CI) | p-value | Absolute mean difference in scores in % (95% CI) | p-value | Absolute mean difference in scores in % (95% CI) | p-value |
| **Education** | Secondary /  high school (v none / primary) | 7.45  (4.07, 10.84) | <0.0001 | 7.07  (3.74, 10.40) | <0.0001 | 6.27  (3.74, 8.79) | <0.0001 | 4.88  (2.69, 7.07) | <0.0001 | 2.55  (-0.39, 5.49) | 0.04 | 7.21  (4.75, 9.68) | <0.0001 |
|  | Technical / University | 7.09  (3.71, 10.48) |  | 7.59  (4.26, 10.92) |  | 7.10  (4.57, 9.62) |  | 6.07  (3.88, 8.26) |  | 4.38  (1.44, 7.32) |  | 15.12  (12.65, 17.58) |  |
|  | Per level increase | 2.25  (0.86, 3.63) | 0.002 | 2.60  (1.24, 3.96) | 0.0002 | 2.66  (1.63, 3.68) | <0.0001 | 2.34  (1.45, 3.23) | <0.0001 | 1.82  (0.63, 3.01) | 0.003 | 5.83  (4.84, 6.82) | <0.0001 |
| **Residential area**** | Rural (v urban) | -3.55  (-7.84, 0.74) | 0.11 | -2.91  (-7.00, 1.18) | 0.16 | -4.18  (-7.40, -0.97) | 0.01 | -2.92  (-5.91, 0.07) | 0.06 | -1.76  (-5.71, 2.20) | 0.39 | -4.88  (-7.95, -1.81) | 0.002 |
| **Marital status** | Unmarried (v married) | -3.39  (-6.47, -0.31) | 0.03 | -4.24  (-7.26, -1.21) | 0.01 | -2.93  (-5.24, -0.62) | 0.01 | -3.82  (-5.81, -1.83) | 0.0002 | -5.80  (-8.43, -3.17) | <0.0001 | -6.12  (-8.44, -3.80) | <0.0001 |
| **No. children at home** | Per child increase | -0.61  (-1.30, 0.07) | 0.08 | -0.59  (-1.27, 0.08) | 0.09 | -0.17  (-0.69, 0.35) | 0.52 | 0.04  (-0.40, 0.49) | 0.85 | 0.19  (-0.40, 0.78) | 0.54 | -0.31  (-0.84, 0.21) | 0.24 |
| **HIV status***** | Positive (v negative / unknown) | -3.65  (-8.65, 1.35) | 0.15 | -2.32  (-7.28, 2.63) | 0.36 | -0.93  (-4.77, 2.92) | 0.64 | -1.99  (-5.08, 1.11) | 0.21 | -2.81  (-6.96, 1.34) | 0.19 | -5.14  (-9.16, -1.11) | 0.01 |
| **BMI** (Kg/m^2^) | <18.5 (v [18.5-25[) | -3.60  (-11.60, 4.40) | 0.10 | -4.90  (-12.78, 2.97) | 0.047 | -0.32  (-6.35, 5.70) | 0.55 | -3.09  (-8.28, 2.11) | 0.02 | -2.75  (-9.62, 4.12) | 0.01 | -5.55  (-11.61, 0.51) | <0.0001 |
|  | [25-30[ | 3.61  (0.16, 7.05) |  | 2.80  (-0.59, 6.19) |  | 1.60  (-0.99, 4.19) |  | 2.82  (0.58, 5.05) |  | 4.02  (1.06, 6.98) |  | 2.09  (-0.52, 4.70) |  |
|  | 30+ | 1.29  (-2.21, 4.79) |  | 3.65  (0.21, 7.10) |  | 1.61  (-1.03, 4.24) |  | 1.91  (-0.37, 4.18) |  | 4.14  (1.13, 7.15) |  | 5.38  (2.73, 8.03) |  |
|  | Per 5 Kg/m^2^ increase | 0.79  (-0.38, 1.96) | 0.18 | 1.36  (0.21, 2.51) | 0.02 | 0.35  (-0.52, 1.23) | 0.43 | 0.69  (-0.07, 1.45) | 0.07 | 1.61  (0.61, 2.61) | 0.002 | 2.17  (1.29, 3.05) | <0.0001 |
| **Other comorbidities** | Any (v none) | -5.68  (-8.60, -2.75) | 0.0002 | -3.12  (-6.02, -0.22) | 0.04 | -3.09  (-5.29, -0.89) | 0.01 | -2.40  (-4.31, -0.49) | 0.01 | 0.81  (-1.73, 3.35) | 0.53 | 0.39  (-1.86, 2.65) | 0.73 |
| **Tobacco (smoking / smokeless)****** | Ever user (v never) | -6.17  (-11.14, -1.20) | 0.02 | -3.32  (-8.49, 1.84) | 0.21 | -6.41  (-10.12, -2.69) | 0.001 | -4.72  (-7.59, -1.86) | 0.001 | -0.67  (-4.57, 3.22) | 0.74 | -4.23  (-8.28, -0.19) | 0.04 |
| **Alcohol** | Ever drunk (v never) | 0.30  (-2.56, 3.15) | 0.84 | 1.45  (-1.37, 4.26) | 0.31 | 0.92  (-1.22, 3.06) | 0.40 | 0.35  (-1.51, 2.20) | 0.71 | 1.50  (-0.95, 3.96) | 0.23 | 0.68  (-1.50, 2.86) | 0.54 |

BMI = Body Mass Index; 95% CI = 95% confidence interval; QoL = Quality of life.

NB : Absolute mean difference in scores in percentage points (95% CI) obtained from linear models adjusted for interviewer, country (Namibia, Nigeria, Uganda), race (Black, non-Black), group of women (breast cancer survivors, cancer-free women), and age (<45, 45-49, 50-54, 55-59, 60-64, and 65+ years).

*Social relationships domain scores were calculated without taking sexual life satisfaction (Q21) into account, due to a high non-response rate to this question.

**Analysis restricted to Uganda and Nigeria as almost all Namibian cancer-free women were living in an urban area.

***Analysis restricted to Namibia and Uganda because only two Nigerian women were HIV-positive.

****Analysis restricted to Namibia as only five Ugandan women and none from Nigeria were tobacco users.

# **Additional Figure 2. Adjusted mean differences (AMD) in scores between breast cancer survivors (BCS) and cancer-free (CF) women for the overall QoL item of the WHOQOL-BREF, before and after further adjustment for correlates**

AMD: Adjusted mean difference; BCS = Breast cancer survivors; BMI = Body mass index; CF = Cancer-free women; 95% CI = 95% confidence interval; QoL = Quality of life.

NB1 : General model (i.e., adjusted for design variables only): Absolute BCS-CF adjusted mean difference (AMD) in QoL scores in percentage points (p.p.), with 95% CI, obtained from linear models adjusted for interviewer (categorical), country (Namibia, Nigeria, Uganda), race (Black, non-Black) and age (<45, 45-49, 50-54, 55-59, 60-64, and 65+ years).

NB2: Adjusted mean scores (95% CI) predicted for a woman in the age category 55-59 years, except for Namibian non-Black women (the age category 60-64 years was used for these women because they were, on average, older than those in other settings), interviewed by the interviewer whose adjusted mean QoL score was closest to the overall adjusted mean QoL score.

# **Additional Figure 3. Adjusted mean differences (AMD) in scores between breast cancer survivors (BCS) and cancer-free (CF) women for the general health QoL item of the WHOQOL-BREF, before and after further adjustment for correlates**

AMD: Adjusted mean difference; BCS = Breast cancer survivors; CF = Cancer-free women; 95% CI = 95% confidence interval.

NB1 & NB2: See footnotes in Additional Figure 2.

NB3: Residential area could not be adjusted for in Namibia as, in this country, cancer-free women where all recruited in an urban area for logistical reasons.

NB4: Tobacco could not be adjusted for in Nigeria and Uganda as prevalence of tobacco use was very low in these two countries (~1%).

# **Additional Figure 4. Adjusted mean differences (AMD) in scores between breast cancer survivors (BCS) and cancer-free (CF) women for the physical health domain of the WHOQOL-BREF, before and after further adjustment for correlates**

AMD: Adjusted mean difference; BCS = Breast cancer survivors; CF = Cancer-free women; 95% CI = 95% confidence interval.

See footnotes in Additional Figures 2 & 3.

# **Additional Figure 5. Adjusted mean differences (AMD) in scores between breast cancer survivors (BCS) and cancer-free (CF) women for the psychological domain of the WHOQOL-BREF, before and after further adjustment for correlates**

AMD: Adjusted mean difference; BCS = Breast cancer survivors; BMI = Body mass index; CF = Cancer-free women; 95% CI = 95% confidence interval.

See footnotes in Additional Figures 2 & 3.

# **Additional Figure 6. Adjusted mean differences (AMD) in scores between breast cancer survivors (BCS) and cancer-free (CF) women for the social relationships (no Q21)* domain of the WHOQOL-BREF, before and after further adjustment for correlates**

AMD: Adjusted mean difference; BCS = Breast cancer survivors; BMI = Body mass index; CF = Cancer-free women; 95% CI = 95% confidence interval.

*Social relationships domain scores were calculated without taking sexual life satisfaction (Q21) into account, due to a high non-response rate to this question.

See footnotes in Additional Figures 2.

# **Additional Figure 7. Adjusted mean differences (AMD) in scores between breast cancer survivors (BCS) and cancer-free (CF) women for the environment domain of the WHOQOL-BREF, before and after further adjustment for correlates**

AMD: Adjusted mean difference; BCS = Breast cancer survivors; BMI = Body mass index; CF = Cancer-free women; 95% CI = 95% confidence intervals.

NB1 to NB4: See footnotes in Additional Figures 2 & 3.

NB5: HIV status could not be adjusted for in Nigeria as prevalence of HIV positivity is very low in this country (<1%).
