# Supplementary material for: Quality of life in long-term breast cancer survivors in Sub-Saharan Africa: the African Breast Cancer–Disparities in Outcomes study
Source: J Cancer Surviv. Author manuscript; Available in PMC 2026 May 6. (PMC12149336; doi:10.1007/s11764-024-01693-1)
Supplement: Supplementary file 1 [file NIHMS2083018-supplement-Supplementary_material.pdf]

Table of Contents

Additional Table 1. Characteristics of women in the ABC-DO cohort in the settings where the QoL study was conducted (i.e., Namibia, Nigeria, and Uganda), by status at the time of the QoL interview 2

Additional Table 2. Correlation* between WHOQOL-BREF General items and QoL domains, and reliability of each QoL domain in the QoL survey 3

Additional Table 3. Mean (95% CI) of domain-specific WHOQOL-BREF QoL percentages in breast cancer survivors and cancer-free women, by country and race 4

Additional Figure 1. Adjusted mean differences (AMD) in WHOQOL-BREF domain scores between breast cancer survivors (BCS) and cancer-free (CF) women among HIV negative women, by country and race 5

Additional Table 4. Correlates of the General QoL items and QoL domains of the WHOQOL-BREF in the QoL survey 6

Additional Figure 2. Adjusted mean differences (AMD) in scores between breast cancer survivors (BCS) and cancer-free (CF) women for the overall QoL item of the WHOQOL-BREF, before and after further adjustment for correlates 7

Additional Figure 3. Adjusted mean differences (AMD) in scores between breast cancer survivors (BCS) and cancer-free (CF) women for the general health QoL item of the WHOQOL-BREF, before and after further adjustment for correlates 8

Additional Figure 4. Adjusted mean differences (AMD) in scores between breast cancer survivors (BCS) and cancer-free (CF) women for the physical health domain of the WHOQOL-BREF, before and after further adjustment for correlates 9

Additional Figure 5. Adjusted mean differences (AMD) in scores between breast cancer survivors (BCS) and cancer-free (CF) women for the psychological domain of the WHOQOL-BREF, before and after further adjustment for correlates 10

Additional Figure 6. Adjusted mean differences (AMD) in scores between breast cancer survivors (BCS) and cancer-free (CF) women for the social relationships (no Q21)* domain of the WHOQOL-BREF, before and after further adjustment for correlates 11

Additional Figure 7. Adjusted mean differences (AMD) in scores between breast cancer survivors (BCS) and cancer-free (CF) women for the environment domain of the WHOQOL-BREF, before and after further adjustment for correlates 12

Additional Table 1. Characteristics of women in the ABC-DO cohort in the settings where the QoL study was conducted (i.e., Namibia, Nigeria, and Uganda), by status at the time of the QoL interview

		Full ABC-DO cohort (n=1333)	BCS (n=357)	Potential BCS but missing (n=110)	p-value*
		N (%)	N (%)	N (%)	
**Country/ethnicity**	Namibia Black	388 (29.1)	136 (85.0)	24 (15.0)	0.001
	Namibia non-Black	114 (8.6)	58 (79.5)	15 (20.5)	
	Nigeria	400 (30.0)	75 (75.8)	24 (24.2)	
	Uganda	431 (32.3)	88 (65.2)	47 (34.8)	
**Age at diagnosis**	Mean age, SD	50.6 (13.6)	51 (12.4)	50 (13.4)	0.34
**Education**	None	155 (11.6)	24 (6.7)	13 (11.8)	0.33
	Primary school	426 (32.0)	96 (26.9)	32 (29.1)	
	Secondary/high school	466 (35.0)	129 (36.1)	39 (35.5)	
	Technical	140 (10.5)	50 (14.0)	14 (12.7)	
	University	146 (11.0)	58 (16.2)	12 (10.9)	
**Residential area***	Urban	683 (51.2)	215 (60.2)	63 (57.3)	0.58
	Rural	650 (48.8)	142 (39.8)	47 (42.7)	
**Comorbidities (at recruitment)**					
**BMI (Kg/m2)**	Mean BMI, SD	26.4 (5.8)	28 (5.9)	26 (5.5)	0.11
**HIV**	Yes	116 (8.7)	33 (9.2)	8 (7.3)	0.52
	No	1217 (91.3)	324 (90.8)	102 (92.7)	
**Other comorbidities**	Yes	708 (53.1)	183 (51.3)	56 (50.9)	0.95
	No	625 (46.9)	174 (48.7)	54 (49.1)	
**BC characteristics**					
**TNM stage at diagnosis**	I	91 (6.8)	42 (11.8)	17 (15.5)	0.03
	II	365 (27.4)	173 (48.5)	35 (31.8)	
	III	604 (45.3)	115 (32.2)	45 (40.9)	
	IV	209 (15.7)	13 (3.6)	6 (5.5)	
	Unknown	64 (4.8)	14 (3.9)	7 (6.4)	
**Tumour subtype**	HR+	421 (31.6)	173 (48.5)	37 (33.6)	0.79
	HR-	158 (11.9)	47 (13.2)	9 (8.2)	
	unknown	754 (56.6)	137 (38.4)	64 (58.2)	
**Any treatment received****	Yes	915 (81.4)	314 (91.3)	83 (79.8)	0.07
	No	134 (11.9)	18 (5.2)	10 (9.6)	
	Unknown	75 (6.7)	12 (3.5)	11 (10.6)	

ABC-DO = African Breast Cancer – Disparities in Outcomes; BC = Breast cancer; BCS = Breast cancer survivors; QoL = Quality of life; SD = Standard deviation.

*p-value obtained from chi-square tests comparing BCS to potential BCS but lost to follow-up. For the BC characteristics, these tests were restricted to non-missing data.

** Among BCS diagnosed with non-metastatic BC who underwent potentially curative treatment: 285 (91%) had surgery (with 81% having mastectomy), 241 (77%) received chemotherapy, 173 (55%) had radiotherapy, and 251 (80%) received endocrine therapy.

Additional Table 2. Correlation* between WHOQOL-BREF General items and QoL domains, and reliability of each QoL domain in the QoL survey

	QOL_{gh}**	QOL_{ov}***	Physical health	Psychological	Social relationships (no Q21)****	Environment	Cronbach α coefficient
**QOL_{gh}****	1	-	-	-	-	-	-
**QOL_{ov}*****	0.72	1	-	-	-	-	-
**Physical health**	0.66	0.59	1	-	-	-	0.86
**Psychological**	0.68	0.67	0.77	1	-	-	0.80
**Social relationships (no Q21)**	0.42	0.43	0.51	0.62	1	-	0.62
**Environment**	0.50	0.54	0.55	0.67	0.59	1	0.85

QoL = Quality of life; QOL_{gh} = General health; QOL_{ov} = Overall QoL.
*Pearson's correlation coefficient.
**two Nigerian BCS had missing QOL_{gh} score.
***Three Nigerian BCS had missing QOL_{ov} score, of which two also had missing QOL_{gh} score.
****Results presented do not take sexual life satisfaction (i.e., Q21) into account, due to the high non-response rate to this question.

Additional Table 3. Mean (95% CI) of domain-specific WHOQOL-BREF QoL percentages in breast cancer survivors and cancer-free women, by country and race

	**Namibia non-Black**		**Namibia Black**		**Nigeria**		**Uganda**		**Overall**	
	**Breast Cancer Survivors (n = 58)**	**Cancer-free women (n = 49)**	**Breast Cancer Survivors (n = 136)**	**Cancer-free women (n = 147)**	**Breast Cancer Survivors (n = 75)**	**Cancer-free women (n = 152)**	**Breast Cancer Survivors (n = 88)**	**Cancer-free women (n = 158)**	**Breast Cancer Survivors (n = 357)**	**Cancer-free women (n = 505)**
QoL Domain	Mean (95%CI)	Mean (95%CI)	Mean (95%CI)	Mean (95%CI)	Mean (95%CI)	Mean (95%CI)	Mean (95%CI)	Mean (95%CI)	Mean (95%CI)	Mean (95%CI)
QOL_{gh} score*	83.6 (78.5, 88.8)	83.2 (77.6, 88.8)	80.0 (76.5, 83.4)	73.5 (70.2, 76.8)	76.3 (71.4, 81.3)	66.8 (63.3, 70.2)	68.5 (63.8, 73.2)	65.3 (61.8, 68.8)	77.0 (74.7, 79.2)	69.9 (67.9, 71.8)
QOL_{ov} score**	84.9 (80.1, 89.8)	79.1 (73.8, 84.4)	82.4 (78.7, 86.0)	64.6 (61.1, 68.1)	80.4 (75.5, 85.3)	65.6 (62.2, 69.1)	71.0 (66.8, 75.3)	66.9 (63.7, 70.1)	79.6 (77.4, 81.8)	67.0 (65.2, 68.9)
Physical health	82.9 (78.8, 87.1)	79.9 (75.4, 84.4)	82.6 (80.0, 85.3)	79.7 (77.2, 82.3)	73.2 (69.4, 77.1)	70.7 (68.0, 73.4)	63.6 (60.0, 67.2)	62.4 (59.7, 65.1)	76.0 (74.1, 77.9)	71.7 (70.1, 73.2)
Psychological	84.0 (81.1, 86.9)	79.5 (76.4, 82.7)	80.9 (78.8, 82.9)	76.3 (74.3, 78.3)	71.8 (68.1, 75.5)	66.1 (63.5, 68.7)	64.8 (61.7, 67.9)	64.2 (61.9, 66.5)	75.5 (73.9, 77.1)	69.8 (68.4, 71.1)
Social relationships (no Q21)	91.8 (88.7, 95.0)	84.4 (81.0, 87.9)	84.7 (82.0, 87.5)	76.7 (74.0, 79.4)	65.0 (59.7, 70.3)	65.7 (62.0, 69.5)	65.8 (61.7, 69.8)	60.0 (57.0, 63.1)	77.1 (74.9, 79.3)	69.0 (67.1, 70.8)
Environment	87.6 (84.2, 91.0)	77.0 (73.3, 80.7)	63.3 (60.4, 66.2)	60.2 (57.4, 63.0)	60.6 (57.4, 63.9)	57.6 (55.3, 59.8)	58.3 (54.5, 62.1)	52.4 (49.6, 55.3)	65.4 (63.5, 67.3)	58.6 (57.0, 60.2)

95% CI = 95% confidence interval; QoL = Quality of life; QOL_{gh} = General health; QOL_{ov} = Overall QoL.
*QOL_{gh} score was missing in two Nigerian BCS;
**QOL_{ov} scores were missing in three Nigerian BCS.

Additional Figure 1. Adjusted mean differences (AMD) in WHOQOL-BREF domain scores between breast cancer survivors (BCS) and cancer-free (CF) women among HIV negative women, by country and race

WHOQOL-BREF domains	N (BCS) / N (CF)	Mean score (95%CI) (BCS)	Mean score (95%CI) (CF)	BCS-CF AMD (95% CI)	p-value
**Overall QOL**					
Namibia non-Black	56/48	86.9 (77.6, 96.2)	81.2 (72.2, 90.2)	5.75 (-1.71, 13.20)	0.13
Namibia Black	118/123	76.4 (68.1, 84.7)	65.0 (56.8, 73.3)	11.34 (4.84, 17.84)	0.001
Namibia Black (urban only)	73/121	77.3 (68.0, 86.7)	63.8 (54.9, 72.7)	13.57 (6.26, 20.87)	0.0004
Nigeria	71/152	83.7 (76.4, 91.0)	68.4 (62.1, 74.7)	15.33 (9.53, 21.14)	<0.0001
Uganda	77/138	76.8 (69.4, 84.2)	67.4 (60.6, 74.2)	9.41 (3.77, 15.05)	0.001
All countries (Black only)	266/413	79.4 (75.0, 83.8)	67.3 (63.3, 71.4)	12.06 (8.62, 15.50)	<0.0001
All countries (all women)	322/461	80.6 (76.6, 84.6)	69.6 (65.8, 73.4)	10.99 (7.80, 14.18)	<0.0001
**General health**					
Namibia non-Black	56/48	86.4 (76.6, 96.2)	86.1 (76.7, 95.6)	0.30 (-7.53, 8.12)	0.94
Namibia Black	118/123	71.8 (63.6, 79.9)	70.5 (62.5, 78.5)	1.28 (-5.07, 7.63)	0.69
Namibia Black (urban only)	73/121	73.7 (64.7, 82.8)	69.4 (60.8, 78.0)	4.36 (-2.70, 11.43)	0.23
Nigeria	71/152	80.1 (72.6, 87.7)	70.1 (63.6, 76.6)	10.06 (4.10, 16.02)	0.001
Uganda	77/138	76.8 (68.8, 84.7)	67.0 (59.7, 74.2)	9.82 (3.77, 15.86)	0.002
All countries (Black only)	266/413	76.8 (72.3, 81.3)	69.7 (65.5, 73.9)	7.14 (3.62, 10.66)	0.0001
All countries (all women)	322/461	77.5 (73.3, 81.6)	71.7 (67.9, 75.6)	5.74 (2.47, 9.00)	0.001
**Physical health**					
Namibia non-Black	56/48	84.4 (76.6, 92.3)	81.3 (73.6, 88.9)	3.17 (-3.12, 9.46)	0.33
Namibia Black	118/123	81.9 (75.7, 88.0)	84.1 (78.0, 90.2)	-2.20 (-7.02, 2.63)	0.37
Namibia Black (urban only)	73/121	81.9 (75.1, 88.7)	83.2 (76.7, 89.6)	-1.27 (-6.55, 4.01)	0.64
Nigeria	73/152	76.8 (71.5, 82.0)	73.0 (68.4, 77.5)	3.80 (-0.34, 7.94)	0.07
Uganda	77/138	66.4 (59.9, 72.9)	65.5 (59.5, 71.4)	0.94 (-3.99, 5.87)	0.71
All countries (Black only)	268/413	75.5 (72.1, 78.9)	74.5 (71.3, 77.6)	1.00 (-1.65, 3.64)	0.46
All countries (all women)	324/461	76.8 (73.6, 79.9)	75.5 (72.6, 78.5)	1.23 (-1.23, 3.69)	0.33
**Psychological**					
Namibia non-Black	56/48	83.9 (78.3, 89.5)	79.4 (74.1, 84.8)	4.48 (0.03, 8.94)	0.052
Namibia Black	118/123	80.9 (76.0, 85.7)	80.5 (75.6, 85.3)	0.41 (-3.40, 4.22)	0.83
Namibia Black (urban only)	73/121	80.9 (75.2, 86.5)	80.0 (74.6, 85.4)	0.83 (-3.61, 5.27)	0.71
Nigeria	73/152	76.8 (71.4, 82.2)	69.9 (65.3, 74.6)	6.84 (2.59, 11.08)	0.002
Uganda	77/138	70.6 (65.3, 75.9)	67.3 (62.4, 72.1)	3.29 (-0.75, 7.33)	0.11
All countries (Black only)	268/413	76.4 (73.4, 79.4)	72.9 (70.1, 75.6)	3.50 (1.16, 5.83)	0.003
All countries (all women)	324/461	77.4 (74.7, 80.1)	74.0 (71.4, 76.5)	3.40 (1.28, 5.52)	0.002
**Social relationships (no Q21)**					
Namibia non-Black	56/48	92.2 (86.3, 98.1)	84.8 (79.1, 90.5)	7.42 (2.74, 12.10)	0.003
Namibia Black	118/123	82.0 (75.4, 88.5)	76.7 (70.3, 83.2)	5.24 (0.13, 10.35)	0.046
Namibia Black (urban only)	73/121	80.5 (72.8, 88.2)	74.5 (67.2, 81.8)	6.00 (0.00, 12.00)	0.051
Nigeria	73/151	65.3 (58.3, 72.3)	66.0 (59.9, 72.0)	-0.67 (-6.20, 4.86)	0.81
Uganda	77/138	72.4 (65.2, 79.6)	66.3 (59.7, 72.9)	6.09 (0.58, 11.60)	0.03
All countries (Black only)	268/412	73.5 (69.5, 77.4)	70.4 (66.8, 74.1)	3.02 (-0.08, 6.13)	0.06
All countries (all women)	324/460	77.0 (73.4, 80.5)	73.1 (69.8, 76.4)	3.84 (1.06, 6.63)	0.01
**Environment**					
Namibia non-Black	56/48	92.4 (86.0, 98.8)	81.2 (75.0, 87.4)	11.16 (6.02, 16.31)	<0.0001
Namibia Black	118/123	63.9 (57.1, 70.7)	65.7 (59.0, 72.5)	-1.86 (-7.19, 3.47)	0.50
Namibia Black (urban only)	73/121	68.0 (60.4, 75.6)	63.6 (56.4, 70.8)	4.42 (-1.52, 10.35)	0.15
Nigeria	73/152	62.8 (58.0, 67.6)	59.6 (55.5, 63.7)	3.21 (-0.55, 6.97)	0.10
Uganda	77/138	63.9 (57.6, 70.3)	55.9 (50.1, 61.7)	8.02 (3.18, 12.85)	0.001
All countries (Black only)	268/413	63.4 (60.0, 66.9)	60.4 (57.2, 63.6)	3.03 (0.34, 5.71)	0.03
All countries (all women)	324/461	66.6 (63.5, 69.8)	62.8 (59.8, 65.7)	3.89 (1.43, 6.36)	0.002

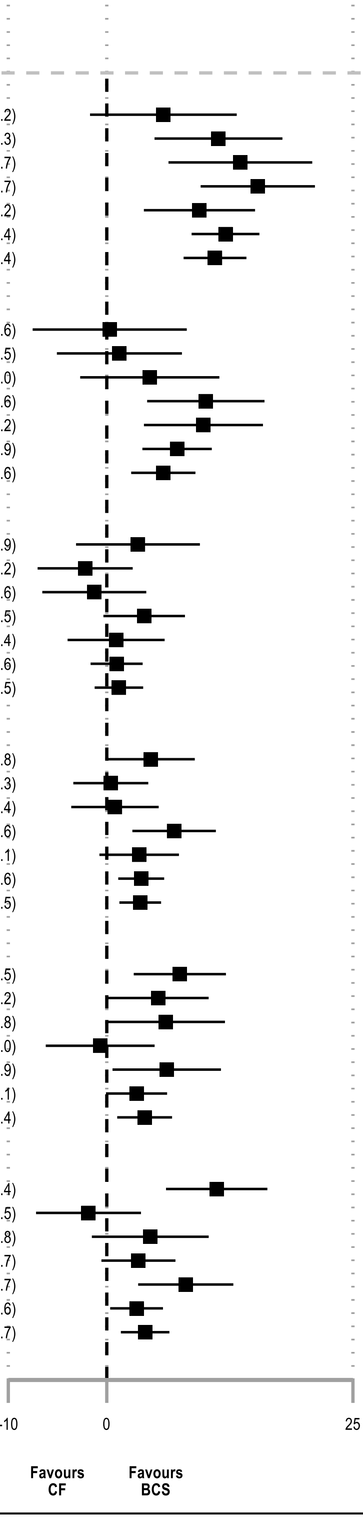

AMD: Adjusted mean difference; BCS = Breast cancer survivors; CF = Cancer-free women; 95% CI = 95% confidence interval; QoL = Quality of life.

NB1: Absolute BCS-CF adjusted mean difference (AMD) in QoL scores in percentage points (p.p.), with 95% CI, obtained from linear models adjusted for interviewer (categorical), country (Namibia, Nigeria, Uganda), race (Black, non-Black) and age (<45, 45-49, 50-54, 55-59, 60-64, and 65+ years).

NB2: Adjusted mean scores (95% CI) predicted for a woman in the age category 55-59 years, except for Namibian non-Black women (the age category 60-64 years was used for these women because they were, on average, older than those in other settings), interviewed by the interviewer whose adjusted mean QoL score was closest to the overall adjusted mean QoL score.

Additional Table 4. Correlates of the General QoL items and QoL domains of the WHOQOL-BREF in the QoL survey

		QOL_{gh}		QOL_{ov}		Physical health		Psychological		Social relationships (no Q21)*		Environment	
		Absolute mean difference in scores in % (95% CI)	p-value	Absolute mean difference in scores in % (95% CI)	p-value	Absolute mean difference in scores in % (95% CI)	p-value	Absolute mean difference in scores in % (95% CI)	p-value	Absolute mean difference in scores in % (95% CI)	p-value	Absolute mean difference in scores in % (95% CI)	p-value
**Education**	Secondary / high school (v none / primary)	7.45 (4.07, 10.84)	<0.0001	7.07 (3.74, 10.40)	<0.0001	6.27 (3.74, 8.79)	<0.0001	4.88 (2.69, 7.07)	<0.0001	2.55 (-0.39, 5.49)	0.04	7.21 (4.75, 9.68)	<0.0001
	Technical / University	7.09 (3.71, 10.48)		7.59 (4.26, 10.92)		7.10 (4.57, 9.62)		6.07 (3.88, 8.26)		4.38 (1.44, 7.32)		15.12 (12.65, 17.58)	
	Per level increase	2.25 (0.86, 3.63)	0.002	2.60 (1.24, 3.96)	0.0002	2.66 (1.63, 3.68)	<0.0001	2.34 (1.45, 3.23)	<0.0001	1.82 (0.63, 3.01)	0.003	5.83 (4.84, 6.82)	<0.0001
**Residential area****	Rural (v urban)	-3.55 (-7.84, 0.74)	0.11	-2.91 (-7.00, 1.18)	0.16	-4.18 (-7.40, -0.97)	0.01	-2.92 (-5.91, 0.07)	0.06	-1.76 (-5.71, 2.20)	0.39	-4.88 (-7.95, -1.81)	0.002
**Marital status**	Unmarried (v married)	-3.39 (-6.47, -0.31)	0.03	-4.24 (-7.26, -1.21)	0.01	-2.93 (-5.24, -0.62)	0.01	-3.82 (-5.81, -1.83)	0.0002	-5.80 (-8.43, -3.17)	<0.0001	-6.12 (-8.44, -3.80)	<0.0001
**No. children at home**	Per child increase	-0.61 (-1.30, 0.07)	0.08	-0.59 (-1.27, 0.08)	0.09	-0.17 (-0.69, 0.35)	0.52	0.04 (-0.40, 0.49)	0.85	0.19 (-0.40, 0.78)	0.54	-0.31 (-0.84, 0.21)	0.24
**HIV status*****	Positive (v negative / unknown)	-3.65 (-8.65, 1.35)	0.15	-2.32 (-7.28, 2.63)	0.36	-0.93 (-4.77, 2.92)	0.64	-1.99 (-5.08, 1.11)	0.21	-2.81 (-6.96, 1.34)	0.19	-5.14 (-9.16, -1.11)	0.01
**BMI** (Kg/m²)	<18.5 (v [18.5-25[)	-3.60 (-11.60, 4.40)		-4.90 (-12.78, 2.97)		-0.32 (-6.35, 5.70)		-3.09 (-8.28, 2.11)		-2.75 (-9.62, 4.12)		-5.55 (-11.61, 0.51)	
	[25-30[	3.61 (0.16, 7.05)	0.10	2.80 (-0.59, 6.19)	0.047	1.60 (-0.99, 4.19)	0.55	2.82 (0.58, 5.05)	0.02	4.02 (1.06, 6.98)	0.01	2.09 (-0.52, 4.70)	<0.0001
	30+	1.29 (-2.21, 4.79)		3.65 (0.21, 7.10)		1.61 (-1.03, 4.24)		1.91 (-0.37, 4.18)		4.14 (1.13, 7.15)		5.38 (2.73, 8.03)	
	Per 5 Kg/m^2 increase	0.79 (-0.38, 1.96)	0.18	1.36 (0.21, 2.51)	0.02	0.35 (-0.52, 1.23)	0.43	0.69 (-0.07, 1.45)	0.07	1.61 (0.61, 2.61)	0.002	2.17 (1.29, 3.05)	<0.0001
**Other comorbidities**	Any (v none)	-5.68 (-8.60, -2.75)	0.0002	-3.12 (-6.02, -0.22)	0.04	-3.09 (-5.29, -0.89)	0.01	-2.40 (-4.31, -0.49)	0.01	0.81 (-1.73, 3.35)	0.53	0.39 (-1.86, 2.65)	0.73
**Tobacco (smoking / smokeless)******	Ever user (v never)	-6.17 (-11.14, -1.20)	0.02	-3.32 (-8.49, 1.84)	0.21	-6.41 (-10.12, -2.69)	0.001	-4.72 (-7.59, -1.86)	0.001	-0.67 (-4.57, 3.22)	0.74	-4.23 (-8.28, -0.19)	0.04
**Alcohol**	Ever drunk (v never)	0.30 (-2.56, 3.15)	0.84	1.45 (-1.37, 4.26)	0.31	0.92 (-1.22, 3.06)	0.40	0.35 (-1.51, 2.20)	0.71	1.50 (-0.95, 3.96)	0.23	0.68 (-1.50, 2.86)	0.54

BMI = Body Mass Index; 95% CI = 95% confidence interval; QoL = Quality of life.

NB : Absolute mean difference in scores in percentage points (95% CI) obtained from linear models adjusted for interviewer, country (Namibia, Nigeria, Uganda), race (Black, non-Black), group of women (breast cancer survivors, cancer-free women), and age (<45, 45-49, 50-54, 55-59, 60-64, and 65+ years).

*Social relationships domain scores were calculated without taking sexual life satisfaction (Q21) into account, due to a high non-response rate to this question.

**Analysis restricted to Uganda and Nigeria as almost all Namibian cancer-free women were living in an urban area.

***Analysis restricted to Namibia and Uganda because only two Nigerian women were HIV-positive.

****Analysis restricted to Namibia as only five Ugandan women and none from Nigeria were tobacco users.

Additional Figure 2. Adjusted mean differences (AMD) in scores between breast cancer survivors (BCS) and cancer-free (CF) women for the overall QoL item of the WHOQOL-BREF, before and after further adjustment for correlates

Models	N (BCS) / N (CF)	Mean score (95%CI) (BCS)	Mean score (95%CI) (CF)	BCS-CF AMD (95% CI)	p-value
**Namibia non-Black**					
Design variables	58/49	87.9 (79.0, 96.9)	81.4 (72.6, 90.2)	6.53 (-0.70, 13.76)	0.08
Design variables + education	58/49	87.3 (78.6, 95.9)	83.0 (74.4, 91.6)	4.26 (-2.93, 11.46)	0.25
Design variables + marital status	58/49	86.8 (77.4, 96.2)	80.8 (71.9, 89.7)	5.97 (-1.39, 13.33)	0.12
Design variables + No. children at home	58/49	87.9 (78.9, 96.9)	81.5 (72.7, 90.4)	6.35 (-1.01, 13.71)	0.09
Design variables + BMI	58/49	86.6 (77.7, 95.6)	80.5 (71.7, 89.3)	6.13 (-1.06, 13.31)	0.10
Design variables + comorbidities	58/49	88.1 (79.1, 97.1)	81.6 (72.8, 90.4)	6.51 (-0.74, 13.77)	0.08
**Namibia Black**					
Design variables	136/147	77.5 (70.1, 84.9)	65.9 (58.6, 73.3)	11.56 (5.35, 17.77)	0.0003
Design variables + education	136/147	77.6 (70.4, 84.9)	65.6 (58.4, 72.8)	12.04 (5.93, 18.14)	0.0001
Design variables + marital status	136/147	77.3 (69.9, 84.7)	66.1 (58.7, 73.5)	11.17 (4.82, 17.52)	0.001
Design variables + No. children at home	136/147	78.1 (70.8, 85.4)	66.5 (59.2, 73.8)	11.62 (5.49, 17.76)	0.0002
Design variables + BMI	136/147	77.6 (70.3, 84.9)	65.3 (58.0, 72.6)	12.35 (6.17, 18.53)	0.0001
Design variables + comorbidities	136/147	77.1 (69.7, 84.5)	66.6 (59.2, 73.9)	10.55 (4.25, 16.85)	0.001
**Namibia Black (urban only)**					
Design variables	82/145	78.4 (69.7, 87.1)	64.2 (56.1, 72.4)	14.19 (7.06, 21.31)	0.0001
Design variables + education	82/145	78.1 (69.5, 86.7)	64.5 (56.4, 72.6)	13.52 (6.45, 20.60)	0.0002
Design variables + marital status	82/145	78.6 (69.8, 87.4)	64.2 (55.9, 72.4)	14.43 (7.08, 21.79)	0.0002
Design variables + No. children at home	82/145	78.6 (70.0, 87.3)	64.6 (56.5, 72.8)	14.00 (6.91, 21.08)	0.0001
Design variables + BMI	82/145	78.1 (69.5, 86.7)	64.3 (56.2, 72.5)	13.76 (6.67, 20.85)	0.0002
Design variables + comorbidities	82/145	78.0 (69.4, 86.7)	65.0 (56.9, 73.2)	13.01 (5.87, 20.15)	0.0004
**Nigeria**					
Design variables	72/152	83.3 (76.0, 90.7)	68.5 (62.1, 74.8)	14.90 (9.12, 20.67)	<0.0001
Design variables + education	72/152	83.4 (76.0, 90.7)	68.4 (62.1, 74.8)	14.92 (9.12, 20.71)	<0.0001
Design variables + marital status	72/152	83.8 (76.5, 91.2)	68.5 (62.2, 74.8)	15.34 (9.55, 21.14)	<0.0001
Design variables + No. children at home	72/152	83.7 (76.4, 91.1)	68.4 (62.1, 74.7)	15.33 (9.51, 21.15)	<0.0001
Design variables + BMI	72/152	84.3 (76.9, 91.7)	68.3 (62.0, 74.6)	16.02 (10.09, 21.96)	<0.0001
Design variables + comorbidities	72/152	83.3 (76.0, 90.7)	68.4 (62.1, 74.8)	14.91 (9.11, 20.71)	<0.0001
**Uganda**					
Design variables	88/157	74.4 (67.7, 81.1)	66.1 (59.9, 72.3)	8.30 (2.95, 13.65)	0.003
Design variables + education	88/157	73.4 (66.6, 80.2)	65.9 (59.7, 72.0)	7.50 (2.10, 12.91)	0.01
Design variables + marital status	88/157	74.3 (67.7, 81.0)	67.1 (60.9, 73.3)	7.27 (1.87, 12.67)	0.01
Design variables + No. children at home	88/157	74.4 (67.7, 81.1)	65.8 (59.5, 72.0)	8.67 (3.28, 14.06)	0.002
Design variables + BMI	88/157	74.6 (67.9, 81.4)	66.1 (59.9, 72.3)	8.54 (3.11, 13.97)	0.002
Design variables + comorbidities	88/157	74.5 (67.8, 81.1)	67.1 (60.9, 73.4)	7.32 (1.90, 12.73)	0.01
**All countries (Black only)**					
Design variables	296/456	78.5 (74.4, 82.6)	67.0 (63.2, 70.8)	11.49 (8.16, 14.82)	<0.0001
Design variables + education	296/456	78.2 (74.1, 82.3)	67.0 (63.2, 70.8)	11.23 (7.91, 14.55)	<0.0001
Design variables + marital status	296/456	78.4 (74.4, 82.5)	67.3 (63.5, 71.1)	11.11 (7.78, 14.44)	<0.0001
Design variables + No. children at home	296/456	78.5 (74.4, 82.6)	67.0 (63.2, 70.8)	11.50 (8.18, 14.82)	<0.0001
Design variables + BMI	296/456	78.8 (74.7, 83.0)	66.7 (62.9, 70.6)	12.10 (8.75, 15.45)	<0.0001
Design variables + comorbidities	296/456	78.5 (74.4, 82.6)	67.4 (63.6, 71.3)	11.03 (7.67, 14.39)	<0.0001
**All countries (all women)**					
Design variables	354/505	79.9 (76.1, 83.6)	69.2 (65.6, 72.8)	10.65 (7.56, 13.74)	<0.0001
Design variables + education	354/505	79.4 (75.6, 83.1)	69.2 (65.6, 72.7)	10.19 (7.12, 13.27)	<0.0001
Design variables + marital status	354/505	79.7 (75.9, 83.5)	69.5 (65.9, 73.1)	10.18 (7.08, 13.28)	<0.0001
Design variables + No. children at home	354/505	79.8 (76.1, 83.6)	69.2 (65.6, 72.8)	10.64 (7.55, 13.73)	<0.0001
Design variables + BMI	354/505	80.1 (76.3, 83.9)	69.0 (65.5, 72.6)	11.08 (7.98, 14.18)	<0.0001
Design variables + comorbidities	354/505	79.8 (76.0, 83.6)	69.6 (66.0, 73.2)	10.18 (7.06, 13.29)	<0.0001

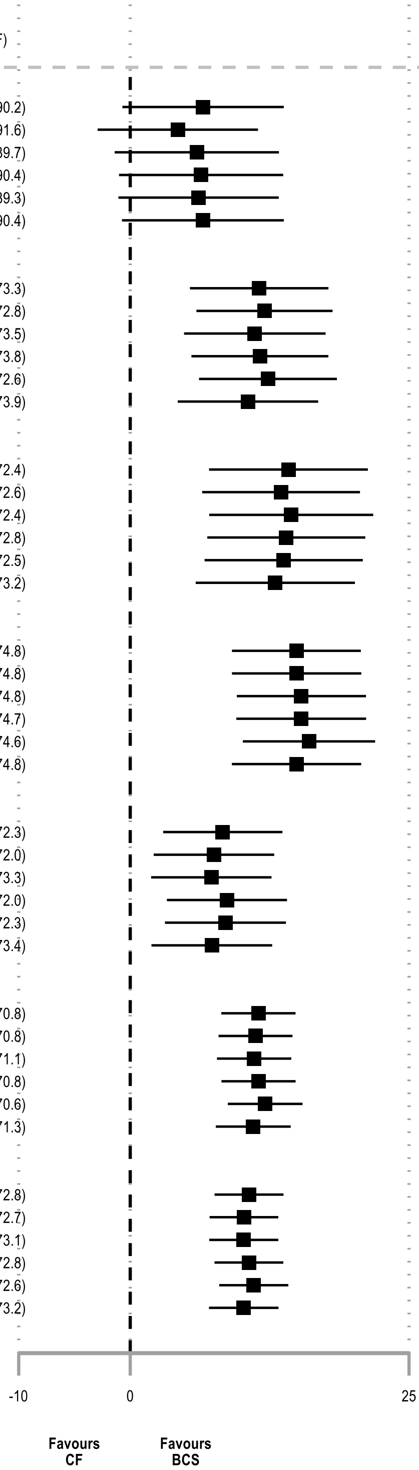

AMD: Adjusted mean difference; BCS = Breast cancer survivors; BMI = Body mass index; CF = Cancer-free women; 95% CI = 95% confidence interval; QoL = Quality of life.

NB1 : General model (i.e., adjusted for design variables only): Absolute BCS-CF adjusted mean difference (AMD) in QoL scores in percentage points (p.p.), with 95% CI, obtained from linear models adjusted for interviewer (categorical), country (Namibia, Nigeria, Uganda), race (Black, non-Black) and age (<45, 45-49, 50-54, 55-59, 60-64, and 65+ years).

NB2: Adjusted mean scores (95% CI) predicted for a woman in the age category 55-59 years, except for Namibian non-Black women (the age category 60-64 years was used for these women because they were, on average, older than those in other settings), interviewed by the interviewer whose adjusted mean QoL score was closest to the overall adjusted mean QoL score.

Additional Figure 3. Adjusted mean differences (AMD) in scores between breast cancer survivors (BCS) and cancer-free (CF) women for the general health QoL item of the WHOQOL-BREF, before and after further adjustment for correlates

Models	N (BCS) / N (CF)	Mean score (95%CI) (BCS)	Mean score (95%CI) (CF)	BCS-CF AMD (95% CI)	p-value
**Namibia non-Black**					
Design variables	58/49	87.8 (78.3, 97.4)	86.1 (76.7, 95.4)	1.79 (-5.92, 9.50)	0.65
Design variables + education	58/49	88.0 (78.1, 97.9)	86.4 (76.8, 95.9)	1.62 (-6.55, 9.80)	0.70
Design variables + marital status	58/49	86.2 (76.3, 96.2)	85.2 (75.7, 94.7)	1.02 (-6.82, 8.85)	0.80
Design variables + No. children at home	58/49	87.7 (78.1, 97.3)	86.3 (76.8, 95.7)	1.44 (-6.40, 9.28)	0.72
Design variables + comorbidities	58/49	87.7 (78.1, 97.2)	85.9 (76.4, 95.3)	1.81 (-5.92, 9.55)	0.65
Design variables + tobacco use	58/49	88.6 (79.4, 97.7)	85.8 (76.8, 94.7)	2.83 (-4.59, 10.24)	0.46
**Namibia Black**					
Design variables	136/147	75.6 (68.6, 82.6)	72.6 (65.6, 79.6)	2.99 (-2.90, 8.88)	0.32
Design variables + education	136/147	76.2 (69.2, 83.1)	72.4 (65.4, 79.3)	3.79 (-2.10, 9.69)	0.21
Design variables + marital status	136/147	75.5 (68.5, 82.6)	72.7 (65.7, 79.7)	2.83 (-3.20, 8.86)	0.36
Design variables + No. children at home	136/147	76.2 (69.2, 83.1)	73.1 (66.2, 80.0)	3.05 (-2.78, 8.88)	0.31
Design variables + comorbidities	136/147	74.8 (68.0, 81.7)	73.8 (67.0, 80.7)	1.03 (-4.85, 6.91)	0.73
Design variables + tobacco use	136/147	75.6 (68.6, 82.6)	72.5 (65.5, 79.5)	3.10 (-2.79, 8.99)	0.30
**Namibia Black (urban only)**					
Design variables	82/145	77.5 (69.4, 85.6)	71.2 (63.6, 78.7)	6.33 (-0.29, 12.95)	0.06
Design variables + education	82/145	77.8 (69.6, 85.9)	71.3 (63.7, 78.9)	6.46 (-0.23, 13.15)	0.06
Design variables + marital status	82/145	77.7 (69.5, 85.9)	71.0 (63.4, 78.7)	6.67 (-0.17, 13.51)	0.06
Design variables + No. children at home	82/145	77.7 (69.6, 85.7)	71.5 (63.9, 79.1)	6.16 (-0.43, 12.75)	0.07
Design variables + comorbidities	82/145	76.8 (69.0, 84.6)	72.5 (65.1, 79.8)	4.34 (-2.12, 10.80)	0.19
Design variables + tobacco use	82/145	77.5 (69.4, 85.6)	71.1 (63.5, 78.8)	6.34 (-0.31, 12.98)	0.06
**Nigeria**					
Design variables	73/152	80.1 (72.5, 87.6)	70.1 (63.6, 76.6)	9.98 (4.06, 15.91)	0.001
Design variables + education	73/152	79.9 (72.3, 87.5)	70.1 (63.6, 76.7)	9.80 (3.86, 15.74)	0.001
Design variables + marital status	73/152	80.4 (72.8, 87.9)	70.1 (63.6, 76.6)	10.25 (4.29, 16.21)	0.001
Design variables + No. children at home	73/152	80.1 (72.5, 87.7)	70.1 (63.6, 76.6)	10.02 (4.04, 16.01)	0.001
Design variables + comorbidities	73/152	80.3 (72.8, 87.8)	70.6 (64.0, 77.1)	9.73 (3.81, 15.66)	0.002
Design variables + residential area	73/152	80.1 (72.6, 87.6)	69.9 (63.4, 76.4)	10.21 (4.26, 16.15)	0.001
**Uganda**					
Design variables	88/157	74.3 (67.1, 81.6)	66.0 (59.3, 72.7)	8.32 (2.54, 14.11)	0.01
Design variables + education	88/157	73.0 (65.6, 80.3)	65.6 (58.9, 72.3)	7.40 (1.46, 13.33)	0.02
Design variables + marital status	88/157	74.3 (67.0, 81.5)	67.1 (60.4, 73.8)	7.21 (1.37, 13.04)	0.02
Design variables + No. children at home	88/157	74.3 (67.1, 81.6)	65.9 (59.2, 72.6)	8.43 (2.59, 14.27)	0.01
Design variables + comorbidities	88/157	74.4 (67.3, 81.6)	67.6 (60.9, 74.3)	6.80 (0.99, 12.61)	0.02
Design variables + residential area	88/157	74.7 (67.4, 82.0)	66.3 (59.7, 73.0)	8.39 (2.61, 14.16)	0.0048
**All countries (Black only)**					
Design variables	297/456	76.9 (72.7, 81.0)	69.8 (66.0, 73.7)	7.04 (3.67, 10.40)	<0.0001
Design variables + education	297/456	76.5 (72.3, 80.6)	69.6 (65.7, 73.4)	6.90 (3.55, 10.25)	0.0001
Design variables + marital status	297/456	76.8 (72.7, 81.0)	70.1 (66.2, 74.0)	6.72 (3.35, 10.10)	0.0001
Design variables + No. children at home	297/456	76.9 (72.7, 81.0)	69.8 (65.9, 73.7)	7.05 (3.69, 10.41)	<0.0001
Design variables + comorbidities	297/456	76.8 (72.7, 80.9)	70.8 (67.0, 74.7)	5.95 (2.58, 9.32)	0.001
Design variables + tobacco use	297/456	76.8 (72.7, 81.0)	69.8 (65.9, 73.6)	7.09 (3.72, 10.45)	<0.0001
Design variables + residential area	297/456	77.3 (73.2, 81.5)	69.6 (65.8, 73.5)	7.71 (4.32, 11.11)	<0.0001
**All countries (all women)**					
Design variables	355/505	77.4 (73.5, 81.2)	71.5 (67.9, 75.1)	5.84 (2.71, 8.98)	0.0003
Design variables + education	355/505	76.9 (73.1, 80.7)	71.3 (67.7, 74.9)	5.57 (2.44, 8.69)	0.001
Design variables + marital status	355/505	77.2 (73.4, 81.1)	71.8 (68.1, 75.4)	5.47 (2.32, 8.61)	0.001
Design variables + No. children at home	355/505	77.3 (73.5, 81.2)	71.5 (67.9, 75.1)	5.84 (2.71, 8.97)	0.0003
Design variables + comorbidities	355/505	77.2 (73.4, 81.1)	72.3 (68.7, 75.9)	4.98 (1.84, 8.12)	0.002
Design variables + tobacco use	355/505	77.5 (73.7, 81.3)	71.5 (67.9, 75.1)	6.02 (2.89, 9.14)	0.0002
Design variables + residential area	355/505	77.7 (73.9, 81.6)	71.3 (67.7, 74.9)	6.44 (3.28, 9.60)	0.0001

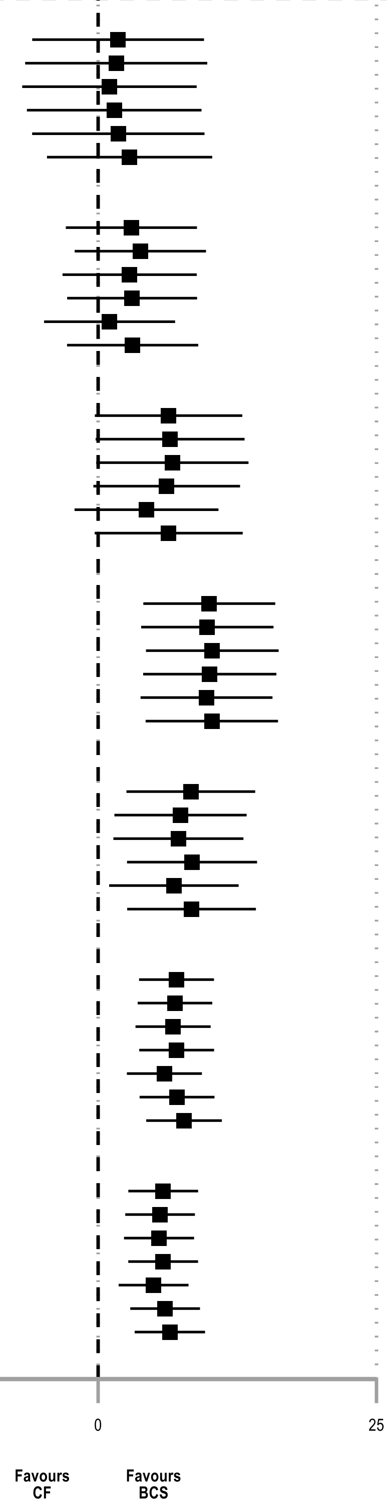

AMD: Adjusted mean difference; BCS = Breast cancer survivors; CF = Cancer-free women; 95% CI = 95% confidence interval.

NB1 & NB2: See footnotes in Additional Figure 2.

NB3: Residential area could not be adjusted for in Namibia as, in this country, cancer-free women where all recruited in an urban area for logistical reasons.

NB4: Tobacco could not be adjusted for in Nigeria and Uganda as prevalence of tobacco use was very low in these two countries (~1%).

Additional Figure 4. Adjusted mean differences (AMD) in scores between breast cancer survivors (BCS) and cancer-free (CF) women for the physical health domain of the WHOQOL-BREF, before and after further adjustment for correlates

Models	N (BCS) / N (CF)	Mean score (95%CI) (BCS)	Mean score (95%CI) (CF)	BCS-CF AMD (95% CI)	p-value
**Namibia non-Black**					
Design variables	58/49	85.5 (78.0, 93.0)	81.7 (74.3, 89.1)	3.81 (-2.30, 9.92)	0.22
Design variables + education	58/49	84.8 (77.0, 92.6)	82.2 (74.7, 89.7)	2.61 (-3.82, 9.04)	0.43
Design variables + marital status	58/49	84.6 (76.7, 92.5)	81.2 (73.7, 88.7)	3.36 (-2.87, 9.58)	0.29
Design variables + comorbidities	58/49	85.7 (78.1, 93.2)	81.9 (74.4, 89.3)	3.79 (-2.33, 9.92)	0.23
Design variables + tobacco use	58/49	86.0 (78.7, 93.3)	81.5 (74.3, 88.7)	4.53 (-1.42, 10.47)	0.14
**Namibia Black**					
Design variables	136/147	82.6 (77.4, 87.8)	84.0 (78.9, 89.2)	-1.48 (-5.84, 2.89)	0.51
Design variables + education	136/147	83.0 (77.9, 88.1)	83.8 (78.7, 88.9)	-0.77 (-5.11, 3.56)	0.73
Design variables + marital status	136/147	82.5 (77.3, 87.7)	84.1 (78.9, 89.3)	-1.57 (-6.04, 2.90)	0.49
Design variables + comorbidities	136/147	82.1 (77.0, 87.2)	84.8 (79.6, 89.9)	-2.66 (-7.05, 1.73)	0.24
Design variables + tobacco use	136/147	82.5 (77.4, 87.7)	83.8 (78.7, 89.0)	-1.30 (-5.63, 3.03)	0.56
**Namibia Black (urban only)**					
Design variables	82/145	82.9 (77.0, 88.7)	83.5 (78.0, 89.0)	-0.68 (-5.47, 4.11)	0.78
Design variables + education	82/145	82.9 (77.0, 88.7)	83.7 (78.3, 89.2)	-0.89 (-5.70, 3.92)	0.72
Design variables + marital status	82/145	83.0 (77.1, 88.9)	83.5 (78.0, 89.0)	-0.50 (-5.45, 4.44)	0.84
Design variables + comorbidities	82/145	82.5 (76.7, 88.2)	84.3 (78.9, 89.7)	-1.86 (-6.60, 2.87)	0.44
Design variables + tobacco use	82/145	82.8 (77.0, 88.7)	83.4 (77.9, 88.9)	-0.55 (-5.34, 4.25)	0.82
**Nigeria**					
Design variables	75/152	76.4 (71.0, 81.7)	73.0 (68.4, 77.6)	3.33 (-0.84, 7.51)	0.12
Design variables + education	75/152	76.2 (70.9, 81.6)	73.2 (68.6, 77.8)	3.05 (-1.11, 7.21)	0.15
Design variables + marital status	75/152	76.7 (71.4, 82.0)	73.1 (68.5, 77.7)	3.62 (-0.56, 7.80)	0.09
Design variables + comorbidities	75/152	76.4 (71.1, 81.8)	73.2 (68.5, 77.8)	3.26 (-0.93, 7.45)	0.13
**Uganda**					
Design variables	88/157	66.6 (60.8, 72.4)	64.8 (59.4, 70.1)	1.87 (-2.73, 6.47)	0.43
Design variables + education	88/157	65.2 (59.5, 71.0)	64.2 (59.0, 69.4)	1.04 (-3.63, 5.71)	0.66
Design variables + marital status	88/157	66.6 (60.8, 72.3)	65.7 (60.3, 71.0)	0.91 (-3.73, 5.54)	0.70
Design variables + comorbidities	88/157	66.7 (60.9, 72.4)	65.5 (60.1, 70.9)	1.18 (-3.50, 5.85)	0.62
**All countries (Black only)**					
Design variables	299/456	75.6 (72.5, 78.7)	74.4 (71.5, 77.3)	1.25 (-1.26, 3.76)	0.33
Design variables + education	299/456	75.2 (72.1, 78.2)	74.2 (71.3, 77.0)	1.02 (-1.46, 3.50)	0.42
Design variables + marital status	299/456	75.6 (72.5, 78.7)	74.6 (71.7, 77.5)	0.96 (-1.55, 3.48)	0.45
Design variables + comorbidities	299/456	75.6 (72.5, 78.7)	74.9 (72.0, 77.7)	0.72 (-1.80, 3.25)	0.57
Design variables + tobacco use	299/456	75.6 (72.5, 78.7)	74.3 (71.4, 77.1)	1.32 (-1.18, 3.82)	0.30
**All countries (all women)**					
Design variables	357/505	76.9 (74.0, 79.8)	75.4 (72.6, 78.1)	1.52 (-0.82, 3.87)	0.20
Design variables + education	357/505	76.3 (73.5, 79.2)	75.2 (72.5, 77.9)	1.13 (-1.19, 3.45)	0.34
Design variables + marital status	357/505	76.8 (73.9, 79.6)	75.6 (72.9, 78.3)	1.19 (-1.16, 3.55)	0.32
Design variables + comorbidities	357/505	76.8 (73.9, 79.7)	75.8 (73.0, 78.5)	1.06 (-1.31, 3.42)	0.38
Design variables + tobacco use	357/505	77.0 (74.1, 79.9)	75.3 (72.6, 78.0)	1.69 (-0.64, 4.03)	0.16

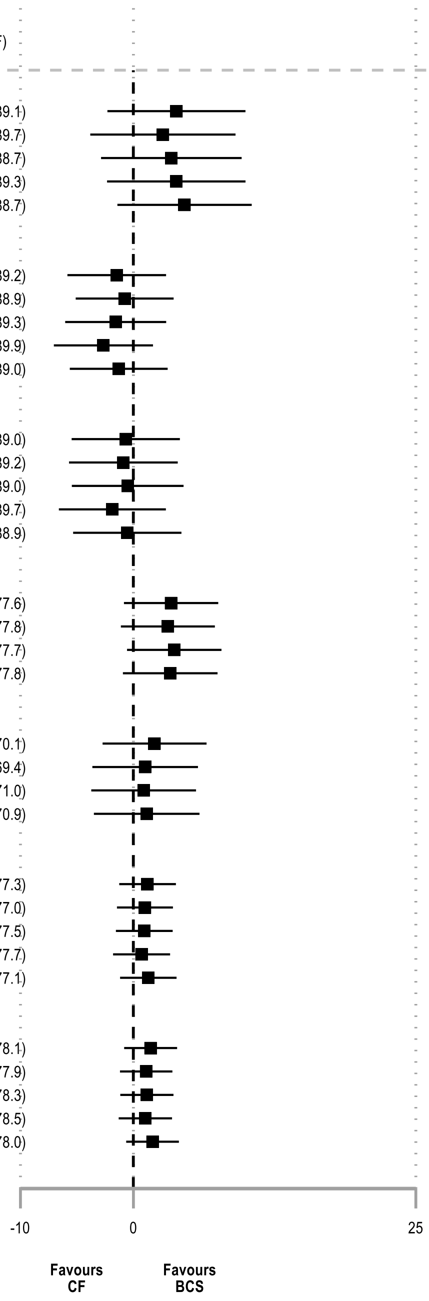

AMD: Adjusted mean difference; BCS = Breast cancer survivors; CF = Cancer-free women; 95% CI = 95% confidence interval.
See footnotes in Additional Figures 2 & 3.

Additional Figure 5. Adjusted mean differences (AMD) in scores between breast cancer survivors (BCS) and cancer-free (CF) women for the psychological domain of the WHOQOL-BREF, before and after further adjustment for correlates

Models	N (BCS) / N (CF)	Mean score (95%CI) (BCS)	Mean score (95%CI) (CF)	BCS-CF AMD (95% CI)	p-value
**Namibia non-Black**					
Design variables	58/49	84.5 (79.2, 89.9)	79.6 (74.3, 84.8)	4.93 (0.61, 9.25)	0.03
Design variables + education	58/49	84.6 (79.2, 89.9)	79.5 (74.2, 84.8)	5.05 (0.60, 9.51)	0.03
Design variables + marital status	58/49	83.3 (77.8, 88.9)	79.0 (73.7, 84.3)	4.35 (-0.02, 8.72)	0.054
Design variables + BMI	58/49	84.1 (78.6, 89.5)	79.2 (74.0, 84.5)	4.80 (0.46, 9.15)	0.03
Design variables + comorbidities	58/49	84.4 (79.1, 89.8)	79.5 (74.2, 84.8)	4.94 (0.60, 9.27)	0.03
Design variables + tobacco use	58/49	84.9 (79.7, 90.1)	79.5 (74.4, 84.6)	5.41 (1.20, 9.63)	0.01
**Namibia Black**					
Design variables	136/147	80.5 (76.4, 84.6)	79.9 (75.8, 84.0)	0.58 (-2.90, 4.06)	0.74
Design variables + education	136/147	80.6 (76.5, 84.6)	79.7 (75.6, 83.7)	0.91 (-2.48, 4.30)	0.60
Design variables + marital status	136/147	80.3 (76.1, 84.4)	80.1 (76.0, 84.3)	0.12 (-3.44, 3.67)	0.95
Design variables + BMI	136/147	80.5 (76.4, 84.6)	79.5 (75.4, 83.6)	1.04 (-2.43, 4.51)	0.56
Design variables + comorbidities	136/147	80.2 (76.1, 84.3)	80.3 (76.2, 84.5)	-0.13 (-3.65, 3.39)	0.94
Design variables + tobacco use	136/147	80.5 (76.4, 84.6)	79.7 (75.6, 83.8)	0.73 (-2.72, 4.18)	0.68
**Namibia Black (urban only)**					
Design variables	82/145	80.6 (75.6, 85.6)	79.4 (74.8, 84.1)	1.15 (-2.92, 5.22)	0.58
Design variables + education	82/145	80.3 (75.4, 85.2)	79.7 (75.1, 84.3)	0.63 (-3.37, 4.62)	0.76
Design variables + marital status	82/145	80.2 (75.2, 85.2)	79.6 (75.0, 84.3)	0.55 (-3.64, 4.74)	0.80
Design variables + BMI	82/145	80.4 (75.5, 85.3)	79.4 (74.8, 84.1)	0.97 (-3.07, 5.02)	0.64
Design variables + comorbidities	82/145	80.4 (75.4, 85.3)	79.9 (75.2, 84.5)	0.51 (-3.58, 4.59)	0.81
Design variables + tobacco use	82/145	80.6 (75.6, 85.5)	79.3 (74.6, 83.9)	1.28 (-2.78, 5.35)	0.54
**Nigeria**					
Design variables	75/152	76.4 (71.0, 81.9)	70.0 (65.3, 74.7)	6.43 (2.20, 10.66)	0.003
Design variables + education	75/152	76.3 (71.0, 81.7)	70.2 (65.6, 74.9)	6.11 (1.92, 10.31)	0.0047
Design variables + marital status	75/152	76.8 (71.4, 82.3)	70.1 (65.4, 74.7)	6.77 (2.54, 11.00)	0.002
Design variables + BMI	75/152	77.3 (71.9, 82.7)	69.8 (65.1, 74.5)	7.50 (3.18, 11.81)	0.001
Design variables + comorbidities	75/152	76.6 (71.2, 82.0)	70.4 (65.7, 75.1)	6.24 (2.02, 10.47)	0.004
Design variables + residential area	75/152	76.5 (71.2, 81.9)	69.6 (65.0, 74.3)	6.89 (2.70, 11.09)	0.002
**Uganda**					
Design variables	88/157	70.6 (65.7, 75.5)	66.7 (62.2, 71.3)	3.86 (-0.05, 7.76)	0.054
Design variables + education	88/157	69.5 (64.5, 74.4)	66.5 (62.1, 71.0)	2.95 (-0.97, 6.86)	0.14
Design variables + marital status	88/157	70.5 (65.7, 75.4)	67.7 (63.2, 72.2)	2.83 (-1.09, 6.74)	0.16
Design variables + BMI	88/157	70.6 (65.7, 75.6)	67.0 (62.5, 71.5)	3.64 (-0.31, 7.60)	0.07
Design variables + comorbidities	88/157	70.6 (65.7, 75.5)	67.3 (62.7, 71.9)	3.31 (-0.66, 7.29)	0.10
Design variables + residential area	88/157	70.6 (65.6, 75.5)	66.7 (62.2, 71.3)	3.85 (-0.06, 7.77)	0.06
**All countries (Black only)**					
Design variables	299/456	76.1 (73.4, 78.9)	72.6 (70.1, 75.2)	3.51 (1.28, 5.74)	0.002
Design variables + education	299/456	75.7 (73.0, 78.5)	72.6 (70.1, 75.1)	3.14 (0.94, 5.33)	0.01
Design variables + marital status	299/456	76.1 (73.3, 78.8)	73.0 (70.4, 75.5)	3.12 (0.90, 5.35)	0.01
Design variables + BMI	299/456	76.3 (73.5, 79.1)	72.5 (69.9, 75.0)	3.83 (1.59, 6.07)	0.001
Design variables + comorbidities	299/456	76.1 (73.3, 78.9)	73.0 (70.5, 75.6)	3.06 (0.81, 5.31)	0.01
Design variables + residential area	299/456	76.4 (73.7, 79.2)	72.5 (69.9, 75.0)	3.94 (1.69, 6.19)	0.001
Design variables + tobacco use	299/456	76.1 (73.4, 78.9)	72.5 (70.0, 75.1)	3.57 (1.35, 5.79)	0.002
**All countries (all women)**					
Design variables	357/505	77.1 (74.6, 79.6)	73.7 (71.3, 76.0)	3.46 (1.43, 5.49)	0.001
Design variables + education	357/505	76.7 (74.2, 79.2)	73.7 (71.4, 76.0)	3.01 (1.00, 5.03)	0.003
Design variables + marital status	357/505	77.0 (74.5, 79.5)	74.0 (71.6, 76.3)	3.03 (1.00, 5.06)	0.004
Design variables + BMI	357/505	77.3 (74.8, 79.7)	73.6 (71.3, 75.9)	3.66 (1.62, 5.70)	0.001
Design variables + comorbidities	357/505	77.1 (74.6, 79.6)	74.0 (71.6, 76.4)	3.10 (1.05, 5.15)	0.003
Design variables + residential area	357/505	77.4 (74.9, 79.9)	73.5 (71.2, 75.9)	3.85 (1.80, 5.90)	0.0002
Design variables + tobacco use	357/505	77.2 (74.8, 79.7)	73.7 (71.3, 76.0)	3.59 (1.57, 5.62)	0.001

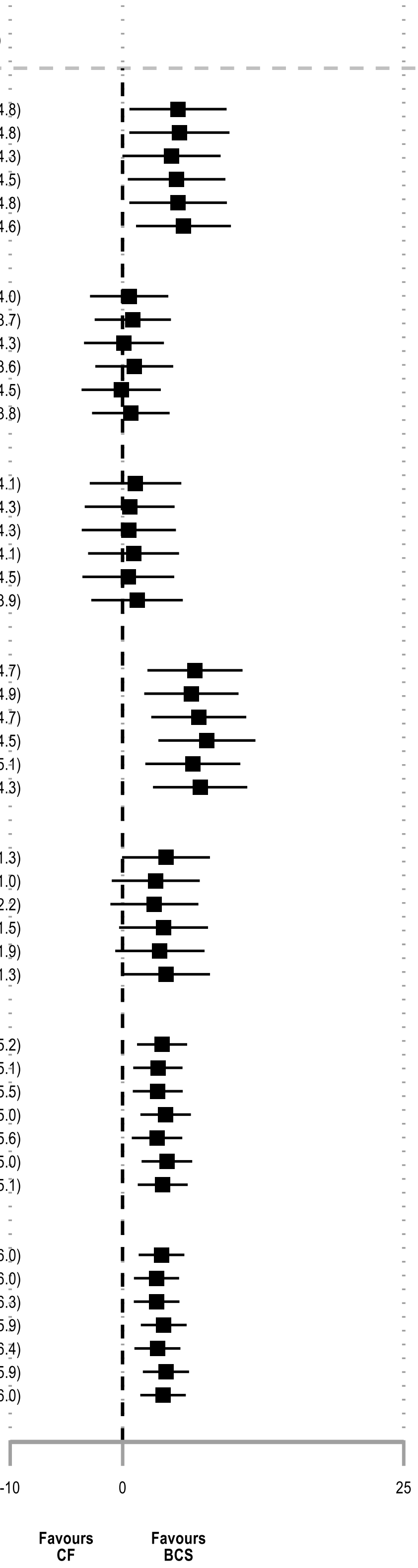

AMD: Adjusted mean difference; BCS = Breast cancer survivors; BMI = Body mass index; CF = Cancer-free women; 95% CI = 95% confidence interval.
See footnotes in Additional Figures 2 & 3.

Additional Figure 6. Adjusted mean differences (AMD) in scores between breast cancer survivors (BCS) and cancer-free (CF) women for the social relationships (no Q21)* domain of the WHOQOL-BREF, before and after further adjustment for correlates

Models	N (BCS) / N (CF)	Mean score (95%CI) (BCS)	Mean score (95%CI) (CF)	BCS-CF AMD (95% CI)	p-value
**Namibia non-Black**					
Design variables	58/49	92.2 (86.5, 97.8)	84.4 (78.9, 89.9)	7.76 (3.20, 12.32)	0.001
Design variables + education	58/49	92.0 (86.3, 97.6)	84.8 (79.2, 90.4)	7.18 (2.50, 11.86)	0.003
Design variables + marital status	58/49	92.4 (86.5, 98.3)	84.5 (78.9, 90.2)	7.87 (3.21, 12.53)	0.001
Design variables + BMI	58/49	92.1 (86.4, 97.8)	84.4 (78.8, 90.0)	7.71 (3.12, 12.29)	0.001
**Namibia Black**					
Design variables	136/147	81.4 (75.7, 87.2)	76.3 (70.6, 82.0)	5.11 (0.27, 9.94)	0.04
Design variables + education	136/147	81.5 (75.9, 87.2)	76.0 (70.4, 81.6)	5.49 (0.74, 10.24)	0.02
Design variables + marital status	136/147	81.0 (75.3, 86.8)	76.7 (71.0, 82.4)	4.31 (-0.61, 9.24)	0.09
Design variables + BMI	136/147	81.5 (75.8, 87.2)	76.1 (70.4, 81.8)	5.42 (0.60, 10.24)	0.03
**Namibia Black (urban only)**					
Design variables	82/145	81.2 (74.2, 88.3)	74.8 (68.3, 81.4)	6.40 (0.66, 12.15)	0.03
Design variables + education	82/145	80.9 (74.0, 87.8)	75.2 (68.7, 81.6)	5.75 (0.08, 11.41)	0.048
Design variables + marital status	82/145	80.4 (73.3, 87.4)	75.3 (68.7, 81.8)	5.10 (-0.78, 10.99)	0.09
Design variables + BMI	82/145	81.0 (74.1, 88.0)	75.1 (68.5, 81.6)	5.95 (0.22, 11.69)	0.04
**Nigeria**					
Design variables	75/151	65.5 (58.5, 72.5)	65.8 (59.8, 71.9)	-0.33 (-5.81, 5.14)	0.91
Design variables + education	75/151	65.5 (58.5, 72.5)	65.9 (59.8, 71.9)	-0.38 (-5.87, 5.12)	0.89
Design variables + marital status	75/151	66.3 (59.3, 73.2)	66.0 (60.0, 71.9)	0.30 (-5.12, 5.73)	0.91
Design variables + BMI	75/151	66.7 (59.7, 73.8)	65.6 (59.6, 71.6)	1.15 (-4.43, 6.73)	0.69
**Uganda**					
Design variables	88/157	70.7 (64.1, 77.3)	64.8 (58.8, 70.9)	5.88 (0.65, 11.12)	0.03
Design variables + education	88/157	70.3 (63.6, 77.0)	64.7 (58.7, 70.8)	5.56 (0.24, 10.88)	0.04
Design variables + marital status	88/157	70.6 (64.1, 77.1)	66.2 (60.2, 72.2)	4.39 (-0.83, 9.62)	0.10
Design variables + BMI	88/157	70.9 (64.3, 77.5)	64.7 (58.6, 70.8)	6.19 (0.90, 11.49)	0.02
**All countries (Black only)**					
Design variables	299/455	73.2 (69.5, 76.9)	70.0 (66.6, 73.4)	3.18 (0.20, 6.16)	0.04
Design variables + education	299/455	72.9 (69.3, 76.6)	70.0 (66.6, 73.4)	2.95 (-0.03, 5.92)	0.053
Design variables + marital status	299/455	73.1 (69.4, 76.7)	70.6 (67.2, 74.0)	2.53 (-0.43, 5.49)	0.09
Design variables + BMI	299/455	73.6 (69.9, 77.3)	69.8 (66.4, 73.2)	3.83 (0.84, 6.83)	0.01
**All countries (all women)**					
Design variables	357/504	76.5 (73.2, 79.8)	72.6 (69.5, 75.7)	3.94 (1.25, 6.63)	0.004
Design variables + education	357/504	76.2 (72.9, 79.5)	72.6 (69.5, 75.7)	3.59 (0.90, 6.28)	0.01
Design variables + marital status	357/504	76.3 (73.0, 79.6)	73.0 (69.9, 76.1)	3.28 (0.60, 5.96)	0.02
Design variables + BMI	357/504	76.8 (73.5, 80.1)	72.3 (69.2, 75.4)	4.48 (1.78, 7.18)	0.001

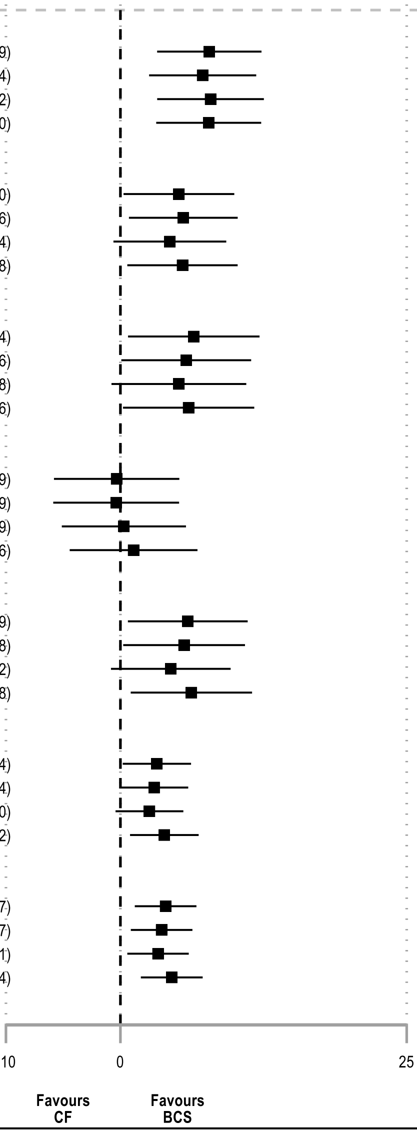

AMD: Adjusted mean difference; BCS = Breast cancer survivors; BMI = Body mass index; CF = Cancer-free women; 95% CI = 95% confidence interval.

*Social relationships domain scores were calculated without taking sexual life satisfaction (Q21) into account, due to a high non-response rate to this question.

See footnotes in Additional Figures 2.

Additional Figure 7. Adjusted mean differences (AMD) in scores between breast cancer survivors (BCS) and cancer-free (CF) women for the environment domain of the WHOQOL-BREF, before and after further adjustment for correlates

Models	N (BCS) / N (CF)	Mean score (95%CI) (BCS)	Mean score (95%CI) (CF)	BCS-CF AMD (95% CI)	p-value
**Namibia non-Black**					
Design variables	58/49	91.2 (85.0, 97.5)	80.4 (74.3, 86.6)	10.77 (5.71, 15.83)	0.0001
Design variables + education	58/49	90.4 (84.8, 96.1)	82.2 (76.6, 87.9)	8.17 (3.47, 12.87)	0.001
Design variables + marital status	58/49	88.8 (82.4, 95.1)	79.2 (73.1, 85.2)	9.58 (4.58, 14.57)	0.0003
Design variables + BMI	58/49	91.3 (85.0, 97.5)	80.4 (74.3, 86.6)	10.82 (5.73, 15.90)	0.0001
Design variables + HIV status	58/49	91.6 (85.4, 97.8)	80.6 (74.5, 86.6)	11.08 (6.07, 16.09)	<0.0001
Design variables + tobacco use	58/49	91.5 (85.3, 97.7)	80.3 (74.3, 86.4)	11.20 (6.19, 16.21)	<0.0001
**Namibia Black**					
Design variables	136/147	64.2 (58.3, 70.1)	65.8 (59.9, 71.7)	-1.56 (-6.53, 3.41)	0.54
Design variables + education	136/147	64.4 (59.3, 69.6)	65.0 (59.9, 70.2)	-0.59 (-4.92, 3.74)	0.79
Design variables + marital status	136/147	63.4 (57.5, 69.2)	66.6 (60.7, 72.4)	-3.18 (-8.18, 1.82)	0.21
Design variables + BMI	136/147	64.4 (58.8, 70.1)	65.2 (59.6, 70.8)	-0.76 (-5.52, 4.01)	0.76
Design variables + HIV status	136/147	64.6 (58.7, 70.6)	66.2 (60.2, 72.1)	-1.54 (-6.50, 3.43)	0.55
Design variables + tobacco use	136/147	64.2 (58.3, 70.1)	65.6 (59.7, 71.4)	-1.40 (-6.34, 3.55)	0.58
**Namibia Black (urban only)**					
Design variables	82/145	69.6 (62.8, 76.4)	64.2 (57.9, 70.5)	5.40 (-0.13, 10.94)	0.06
Design variables + education	82/145	68.8 (62.8, 74.8)	64.9 (59.2, 70.6)	3.89 (-1.06, 8.85)	0.13
Design variables + marital status	82/145	68.3 (61.6, 75.0)	64.8 (58.6, 71.1)	3.43 (-2.18, 9.05)	0.23
Design variables + BMI	82/145	69.2 (62.6, 75.9)	64.6 (58.4, 70.8)	4.65 (-0.79, 10.09)	0.10
Design variables + HIV status	82/145	69.7 (62.9, 76.5)	64.4 (58.0, 70.8)	5.35 (-0.20, 10.90)	0.06
Design variables + tobacco use	82/145	69.5 (62.8, 76.3)	63.9 (57.6, 70.2)	5.65 (0.13, 11.16)	0.046
**Nigeria**					
Design variables	75/152	63.1 (58.3, 67.9)	59.5 (55.3, 63.6)	3.65 (-0.10, 7.41)	0.06
Design variables + education	75/152	63.0 (58.3, 67.7)	59.7 (55.6, 63.8)	3.27 (-0.41, 6.96)	0.08
Design variables + marital status	75/152	63.3 (58.4, 68.1)	59.5 (55.3, 63.6)	3.79 (0.01, 7.56)	0.050
Design variables + BMI	75/152	64.3 (59.6, 69.1)	59.2 (55.1, 63.2)	5.15 (1.39, 8.91)	0.01
Design variables + residential area	75/152	63.2 (58.5, 67.9)	59.1 (55.0, 63.2)	4.14 (0.44, 7.84)	0.03
**Uganda**					
Design variables	88/157	64.4 (58.7, 70.2)	55.5 (50.2, 60.8)	8.92 (4.33, 13.51)	0.0002
Design variables + education	88/157	62.2 (56.5, 67.8)	55.0 (50.0, 60.1)	7.11 (2.65, 11.58)	0.002
Design variables + marital status	88/157	64.3 (58.7, 70.0)	56.6 (51.3, 61.9)	7.79 (3.18, 12.40)	0.001
Design variables + BMI	88/157	64.6 (58.8, 70.4)	55.4 (50.1, 60.7)	9.20 (4.55, 13.84)	0.0001
Design variables + residential area	88/157	64.8 (59.0, 70.6)	55.8 (50.5, 61.1)	8.98 (4.40, 13.55)	0.0002
Design variables + HIV status	88/157	64.8 (59.1, 70.6)	55.9 (50.6, 61.2)	8.90 (4.33, 13.48)	0.0002
**All countries (Black only)**					
Design variables	299/456	63.8 (60.6, 67.0)	60.2 (57.2, 63.2)	3.59 (1.00, 6.18)	0.01
Design variables + education	299/456	63.0 (60.0, 66.0)	60.2 (57.4, 63.0)	2.80 (0.36, 5.23)	0.02
Design variables + marital status	299/456	63.7 (60.6, 66.9)	60.7 (57.7, 63.7)	3.04 (0.46, 5.61)	0.02
Design variables + BMI	299/456	64.5 (61.3, 67.6)	59.9 (56.9, 62.8)	4.60 (2.02, 7.17)	0.001
Design variables + residential area	299/456	64.6 (61.5, 67.8)	59.8 (56.9, 62.7)	4.80 (2.23, 7.37)	0.0003
Design variables + HIV status	299/456	64.1 (60.9, 67.3)	60.4 (57.5, 63.4)	3.65 (1.06, 6.23)	0.01
Design variables + tobacco use	299/456	63.8 (60.6, 67.0)	60.1 (57.2, 63.1)	3.66 (1.07, 6.24)	0.01
**All countries (all women)**					
Design variables	357/505	66.8 (63.8, 69.7)	62.5 (59.7, 65.3)	4.27 (1.88, 6.67)	0.001
Design variables + education	357/505	65.7 (62.9, 68.4)	62.5 (59.9, 65.1)	3.16 (0.93, 5.39)	0.01
Design variables + marital status	357/505	66.5 (63.6, 69.4)	63.0 (60.2, 65.7)	3.58 (1.21, 5.95)	0.003
Design variables + BMI	357/505	67.2 (64.3, 70.1)	62.2 (59.4, 64.9)	4.99 (2.61, 7.37)	<0.0001
Design variables + residential area	357/505	67.5 (64.6, 70.4)	62.1 (59.4, 64.8)	5.36 (2.99, 7.74)	<0.0001
Design variables + HIV status	357/505	67.1 (64.1, 70.0)	62.7 (60.0, 65.5)	4.35 (1.97, 6.74)	0.0004
Design variables + tobacco use	357/505	66.9 (63.9, 69.8)	62.5 (59.7, 65.2)	4.39 (2.00, 6.78)	0.0003

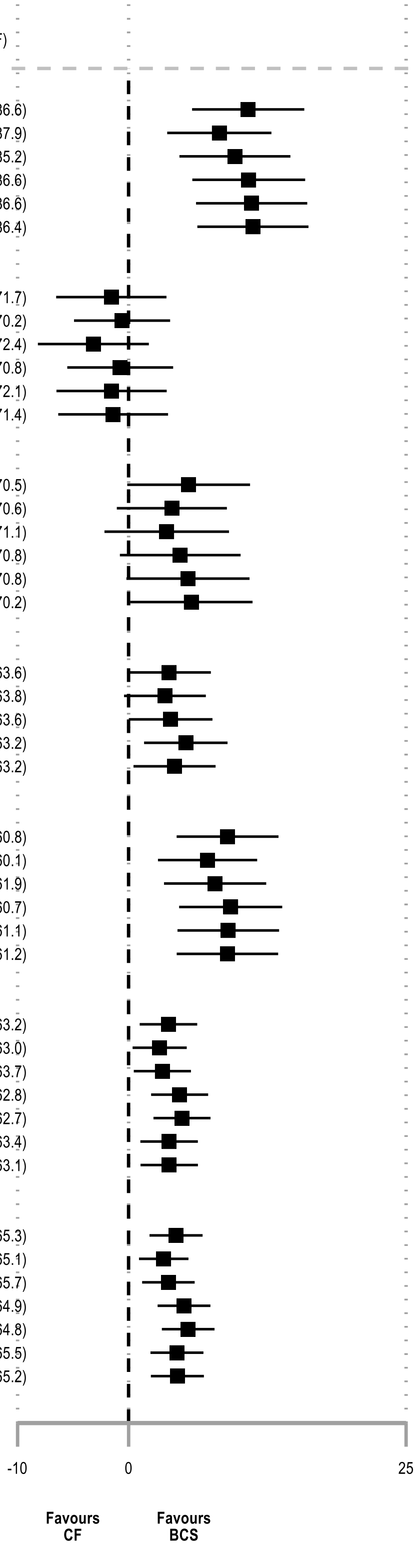

AMD: Adjusted mean difference; BCS = Breast cancer survivors; BMI = Body mass index; CF = Cancer-free women; 95% CI = 95% confidence intervals.

NB1 to NB4: See footnotes in Additional Figures 2 & 3.

NB5: HIV status could not be adjusted for in Nigeria as prevalence of HIV positivity is very low in this country (<1%).
